# Methionine biosynthesis enzyme MoMet2 is required for rice blast fungus pathogenicity by promoting virulence gene expression via reducing 5mC modification

**DOI:** 10.1371/journal.pgen.1010927

**Published:** 2023-09-21

**Authors:** Huimin Li, Pengcheng Mo, Jun Zhang, Zhuoer Xie, Xinyu Liu, Han Chen, Leiyun Yang, Muxing Liu, Haifeng Zhang, Ping Wang, Zhengguang Zhang

**Affiliations:** 1 Department of Plant Pathology, College of Plant Protection, Nanjing Agricultural University, and Key Laboratory of Integrated Management of Crop Diseases and Pests, Ministry of Education, Nanjing, China; 2 The Key Laboratory of Plant Immunity, Nanjing Agricultural University, Nanjing, China; 3 Department of Microbiology, Immunology, and Parasitology, Louisiana State University Health Sciences Center, New Orleans, Louisiana, United States of America; Australian National University Research School of Biology, AUSTRALIA

## Abstract

The emergence of fungicide resistance severely threatens crop production by limiting the availability and application of established fungicides. Therefore, it is urgent to identify new fungicidal targets for controlling plant diseases. Here, we characterized the function of a conserved homoserine O-acetyltransferase (HOA) from the rice blast fungus *Magnaporthe oryzae* that could serve as the candidate antifungal target. Deletion of the *MoMET2* and *MoCYS2* genes encoding HOAs perturbed the biosynthesis of methionine and S-adenyl methionine, a methyl group donor for epigenetic modifications, and severely attenuated the development and virulence of *M*. *oryzae*. The ∆*Momet2* mutant is significantly increased in 5-methylcytosine (5mC) modification that represses the expression of genes required for pathogenicity, including *MoGLIK* and *MoCDH-CYT*. We further showed that host-induced gene silencing (HIGS) targeting *MoMET2* and *MoCYS2* effectively controls rice blasts. Our studies revealed the importance of HOA in the development and virulence of *M*. *oryzae*, which suggests the potential feasibility of HOA as new targets for novel anti-rice blast measurements.

## Author summary

Despite the effectiveness of available antifungal agents in combating rice blasts, the emergence of resistance to these agents requires the reexamination of fungal pathogens, plant-fungi interactions, and virulence factors. Characterization of virulence factors is expected to improve the understanding of fungus pathogenesis and to help exploration for an antifungal drug target. We here reported that HOAs of the fungal original could serve as a potential target for antifungal agents. Specifically, we found that homoserine O-acetyltransferases MoMet2 and MoCys2 are important in the biosynthesis of methionine and S-adenyl methionine that are critical for the growth, development, and virulence of *M*. *oryzae*. We further demonstrated that MoMet2-mediated 5mC modification regulates gene expression and is required for infection by the rice blast fungus. Additionally, using host-induced gene silencing (HIGS) targeting *MoMET2* and *MoCYS2* genes provided protection against rice blasts. In all, the studies shed significant new insights in understanding the role of methionine synthesis in fungal pathogenicity and in identifying potential antifungal targets.

## Introduction

Rice blast caused by *Magnaporthe oryzae* results in over 10% yield loss worldwide [1,2]. To combat this disease, several control measures have been developed, including the most widely used resistant cultivars and fungicides [3,4]. However, resistance to disease can be easily lost following continuous cultivation due to fungal adaptability and rapid variation, as well as rapidly developed resistance to fungicides [4]. Therefore, it is urgent to develop novel disease control strategies.

In eukaryotes, DNA methylation plays a key role in cellular activities, including genome regulation and development [5–7]. This process is catalyzed by a family of DNA methyltransferases (DNMTs) that transfer a methyl group from S-adenyl methionine (SAM) to DNA, where it is established and maintained [8]. DNA cytosine methylation (5-methylcytosine, 5mC), which occurs in diverse eukaryotic organisms, including animals, plants, and fungi [9], is the first discovered and most extensively studied DNA methylation. 5mC is involved in the silencing of transposable elements (TEs) and repeat sequences, genome stability, development, and transcription regulation [10–12].

At present, genome-wide DNA methylation examined in several fungi using bisulfite sequencing indicates the global DNA methylation level is very low compared to the higher eukaryotic counterparts [13,14]. DNA methylation patterns in fungi such as *Beauveria bassiana*, *Cryphonectria parasitica*, *Mucor rouxii*, *Yarrowia lipolytica*, and *Ustilago maydis* are closely related with the developmental stages [15,16]. Methylomes assays showed that 0.22 and 1.5% cytosines in the genomes of *M*. *oryzae* and *Neurospora crassa* are methylated, respectively. This methylation contributes to gene expression, transposon silencing, and heterochromatin formation [17–19]. However, fungal genome-wide DNA methylation during plant-fungi interactions has not been reported, and there are few reports to date about methylated promoters mediating gene transcription in fungi.

Various DNMTs are involved in the establishment and maintenance of DNA methylation, but the distributions and numbers of enzymes involved are highly variable [20]. DNMTs involve numerous pathways and methionine (Met) biosynthetic pathways are often focused on because they provide Met for generating SAM, an important methyl donor for cellular methylation including DNA methylation. Incidentally, the Met biosynthetic pathways are absent in mammals, making them attractive targets for antimicrobial compound discovery [21].

The canonical Met biosynthetic pathway consists of a sulfur uptake pathway and Met biosynthetic pathways A and B [22]. Met can only be synthesized by pathway A in plants and some bacteria, whereas it is synthesized by both pathways A and B in fungi. This suggests that pathway B is specifically synthetic to fungi [23,24]. Homoserine O-acetyltransferases (HOAs) catalyze the formation of l-O-acetyl-homoserine from l-homoserine through the transfer of an acetyl group from acetyl-CoA, and they function as the first committed step required for Met biosynthesis pathway B [25,26]. Additionally, HOAs are crucial for the synthesis of Met and cysteine (Cys) in the yeast *Saccharomyces cerevisiae*, and the loss of HOA function results in Met auxotrophs in *Candida albicans*, *Candida guilliermondii*, and *S*. *cerevisiae* [27–30]. Given the importance of HOA in cellular activities, small molecule inhibitors have been developed that inhibit HOA enzyme activities in human pathogenic fungi [31,32]. Interestingly, the loss of HOA function decreases the pathogenicity of *Gibberella zeae* to wheat and maize [33]. These studies implied that HOAs could be potential candidates for fungicide targets to control fungal diseases. To examine if HOAs could serve as potential fungicide targets, we characterized the function of HOAs encoded by *MoMET2* and *MoCYS2* in *M*. *oryzae*. Further, as host-induced gene silencing (HIGS) and small molecule inhibitor screening are two recently developed approaches to target fungal virulence genes and have the potential to be utilized as the new approaches for managing diseases, we employed HIGS targeting *MoMET2* and *MoCYS2* that effectively controlled the rice blast. Our studies revealed the importance of HOA enzymes in *M*. *oryzae* and demonstrated their great potential as fungicidal targets.

## Results

### Identification of HOA encoding genes in *M*. *oryzae*

**Fig 1:**
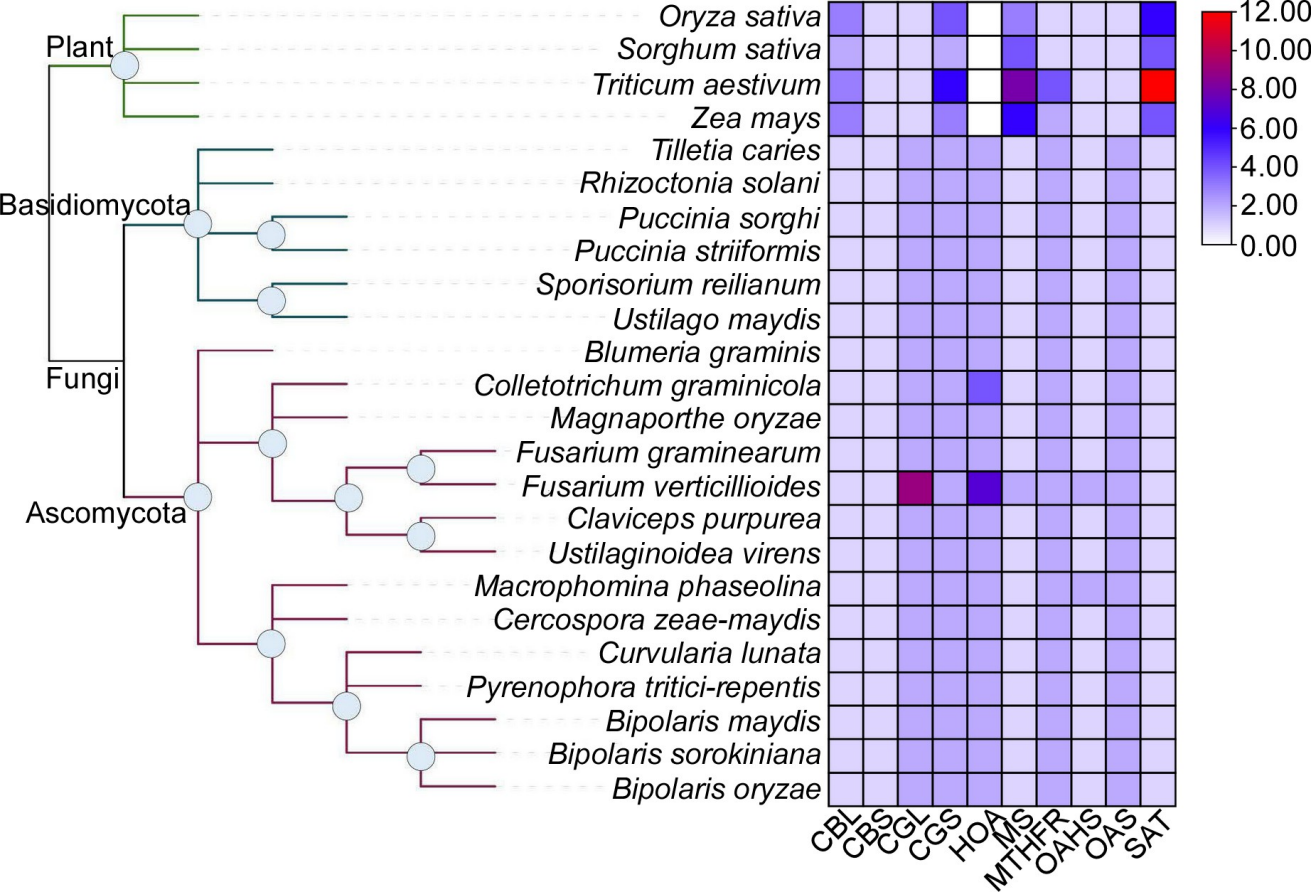
HOAs are present in diverse fungal pathogens but absent in their gramineous hosts. The heat map shows the gene number of the key enzymes in the biosynthesis of methionine in fungi and plants examined. CBL, cystathionine β-lyase. CBS, cystathionine β-synthase. CGL, cystathionine γ-lyase. CGS, cystathionine γ-synthase. HOA, homoserine O-acetyltransferase. MS, methionine synthase. MTHFR, methylene tetrahydrofolate reductase. OAHS, O-acetylhomoserine synthase. OAS, O-acetylserine sulfhydrylase. SAT, serine acetyltransferase.

To explore novel fungicidal targets in *M*. *oryzae*, we focused on key enzymes in the Met biosynthesis pathways. Using BlastP to search homologous protein sequences, we discovered potential HOA homologs that catalyze the first committed step in Met biosynthesis [26] and found that these Met synthetases are specific to fungi and not present in cereal crops (Fig 1).

In *S*. *cerevisiae*, the Met HOA encoding gene *ScMET2* has been extensively studied previously [34]. Using *ScMET2* as a tracer to BLAST the genome of *M*. *oryzae* (http://fungidb.org/fungidb/), we identified two putative Met2 proteins, MGG_01469 and MGG_14202. Sequence alignments showed that MGG_01469 and MGG_14202 share 44.86% and 20.55% amino acid identity, respectively, with ScMet2 (S1A Fig). MGG_14202 also shared 70.27% identity to the putative serine-O-acetyltransferase Cys2 protein in *Colletotrichum chlorophyti* (S1B Fig). Therefore, we named MGG_01469 gene as *MoMET2* and MGG_14202 gene as *MoCYS2*. Pfam domain analysis indicated that MoMet2 and MoCys2 contain the Abhydrolase_1 domain (S2B Fig). Additionally, phylogenetic analysis showed that MoMet2 and MoCys2 are well conserved among Ascomycetes (S2A Fig).

### MoMet2 and MoCys2 are required for vegetative growth, conidiation, and virulence of *M*. *oryzae*

To examine the functions of MoMet2 and MoCys2, we generated individual ∆*Momet2* and ∆*Mocys2*, and double Δ∆*Mocys2Momet2* mutant strains (S3 Fig). The individual Δ*Momet2* and ∆*Mocys2* mutants displayed apparent defects in vegetative growth and conidiation in comparison to the wild-type (WT) strain (S2 Table). Both ∆*Momet2* and Δ∆*Mocys2Momet2* mutants failed to produce conidia on straw decoction and corn (SDC) medium, but the Δ*Momet2* mutant could still produce conidia on complete medium (CM) (S2 Table). The defect phenotypes were restored in the Δ*Momet2* and ∆*Mocys2* mutants complemented with WT *MoMET2* and *MoCYS2*, respectively. These results indicated that MoMet2 and MoCys2 are required for vegetative growth and conidiation.

**Fig 2:**
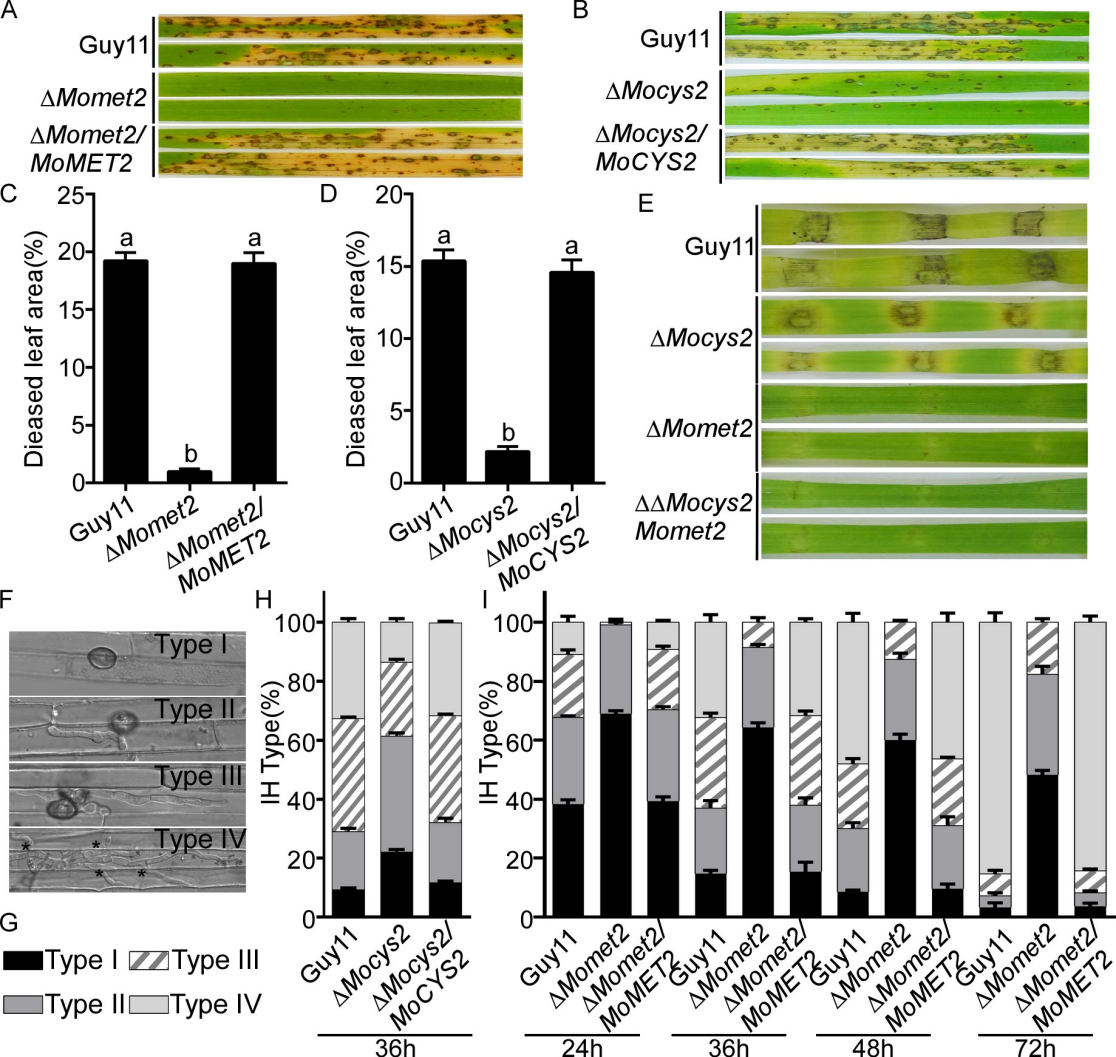
MoMet2 and MoCys2 are important for virulence in *M*. *oryzae*. (A, B) Pathogenicity assay. Four milliliters of conidial suspension (5 × 10^4^ spores ml^-1^) of each strain were used for spraying on *Oryza sativa* cv. CO39 and photographed 7 dpi. (C, D) Diseased leaf area analysis. Data are presented as a bar chart showing the percentage of lesion area analyzed by ImageJ. Error bars represent the SD of three biological replicates and different letters indicate significant differences (*P* < 0.05) tested by one-way ANOVA with Duncan’s post hoc test. (E) Pathogenicity test on barley leaves. Unwounded barley leaves were incubated with mycelial pellets of different strains. Photographs were taken at 5 dpi. (F, G, H, I) Statistical analysis for each type of invasive hyphae (IH) shape. Type I, no penetration; type II, only with a single IH without branches; type III, with 1–3 branches but restricted in one cell; type IV, more than three branches and extended to neighboring plant cells. For each tested strain, 100 infecting hyphae (n = 100) were counted per replicate, and the experiment was repeated three times with similar results. Bar = 10 μm.

To examine the role of *MoMET2* and *MoCYS2* in fungal pathogenicity, we inoculated conidial suspensions of WT, Δ*Momet2*, Δ*Mocys2*, and complementation strains on the susceptible rice cultivar CO39 and barley seedling leaves. The Δ*Momet2* and Δ*Mocys2* mutants were significantly reduced in the infection of rice (Fig 2A, 2B, 2C and 2D). Similar results were observed on the detached barley cultivar four-arris leaves (Fig 2E). As the Δ∆*Mocys2Momet2* double mutant could not produce conidia, mycelia of WT, Δ*Momet2*, Δ*Mocys2*, and Δ∆*Mocys2Momet2* were used to inoculate the detached barley seedling leaves. The Δ*Mocys2* mutant exhibited very restricted lesions on unwounded leaves, while the Δ*Momet2* and Δ∆*Mocys2Momet2* mutants displayed no obvious lesions (Fig 2E). These results demonstrated that both MoMet2 and MoCys2 are required for the virulence of *M*. *oryzae*, with MoMet2 having a stronger role.

To fully assess virulence attenuation, we performed an infection assay on rice sheath and observed invasive hyphae (IH) growth at 100 appressorium penetration sites at 24, 36, 48, and 72 h post-inoculation (hpi). We rated the hyphal growth as type I (no penetration), type II (with primary IH), type III (secondary IH does not extend to neighboring plant cells), and type IV (IH extended into neighboring plant cells) (Fig 2F and 2G) [35]. WT and complementation strains exhibited approximately 90% successful appressorium penetration events, with more than 70% type III and IV IH at 36 hpi (Fig 2H). In contrast, only 78% Δ*Mocys2* appressoria had successful penetration with less than 40% of penetration sites showing type III and IV IH growth (Fig 2H). Strikingly, the Δ*Momet2* mutant was restricted to types I, II, and III in IH growth at 24, 36, 48, and 72 hpi within one cell (Fig 2I). These results indicated that MoMet2 and MoCys2 play an important role in IH growth and host colonization.

To test whether MoMet2 and MoCys2 share the conserved HOA function, we expressed both proteins in *S*. *cerevisiae* HOA homolog mutant (∆*Scmet2*) lacking HOA [36], and found that the co-expression of *MoMET2* and *MoCYS2*, but not individually, rescues the growth defect (S4 Fig). Meanwhile, ScMet2 could partially rescue the growth defect of the Δ*Momet2* mutant on CM and the Δ*Mocys2* mutant on CM, minimal medium (MM), and SDC (S5 Fig). These results indicated that MoMet2 and MoCys2 are functionally similar to ScMet2.

### Exogenous Met or Cys partially rescue mutants’ defects in vegetative growth, conidiation, and virulence

The Δ*Momet2* and Δ∆*Mocys2Momet2* mutants showed sparse mycelial growth on CM and SDC media but not at all on MM (S6A Fig and S2 Table). However, growth was restored in both mutants if MM was supplemented with 1.0 mM Met or 1.0 mM Cys (S6A Fig). The Δ*Momet2* and Δ*Mocys2* mutants produced dense and dark aerial hyphae, in contrast to the sparsely grown hyphae on CM without exogenous amino acids (S6A Fig). In addition, the Δ*Momet2* mutant failed to produce any conidia on SDC medium (S6B and S6C Fig), which was rescued by adding 1.0 mM Met (S6B and S6D Fig). Interestingly, 1.0 mM Cys could partially rescue the Δ*Mocys2* mutant in growth but not conidia production. These data validated that Δ*Momet2* and Δ*Mocys2* are auxotrophic mutants.

Moreover, we examined whether virulence defects of the mutants could be restored by exogenous Met or Cys. Conidia of the Δ*Momet2* and Δ*Mocys2* mutants were supplemented with 50 mM Met or 1.0 mM Cys before inoculating on the detached barley leaves. The Δ*Momet2* mutant formed typical blast lesions in the presence of 50 mM Met (S6E Fig). Similar results were observed for the Δ*Mocys2* mutant supplemented with exogenous 1.0 mM Cys (S6F Fig). Taken together, these results suggested that exogenous Met or Cys could rescue virulence defects of the mutants.

### Deletion of MoMet2 and MoCys2 decreases Met and SAM biosynthesis, but only MoMet2 has an impact on 5mC

**Fig 3:**
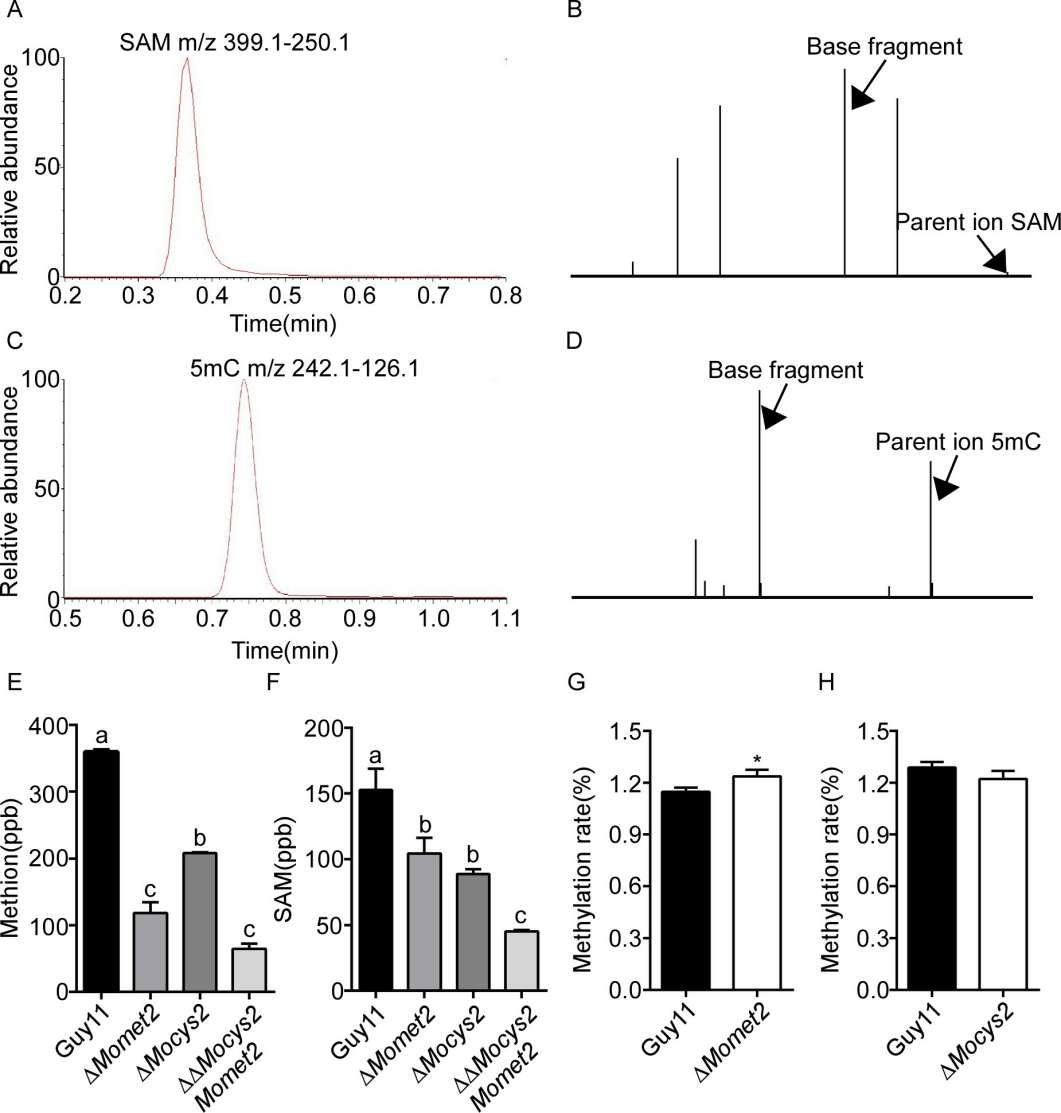
*MoMET2* is involved in 5mC DNA methylation in *M*. *oryzae*. (A) *M*. *oryzae* mycelium SAM was detected by UPLC. The selective multiple reaction monitoring (MRM) transitions for SAM were set as m/z 399.1–250.1. Peak diagram of *M*. *oryzae* mycelium. (B) *M*. *oryzae* SAM are detected by UPLC-ESI-MS/MS. The parent ion SAM (m/z near 399.1) and base fragment (m/z near 250.1) highlighted by arrows from samples also match standard SAM. (C) *M*. *oryzae* mycelium 5mC was detected by UPLC. The selective multiple reaction monitoring (MRM) transitions for 5mC were set as m/z 242.1–126.1. Peak diagram of *M*. *oryzae* gDNA samples. (D) *M*. *oryzae* 5mC is detected by UPLC-ESI-MS/MS. The parent ion 5mC (m/z near 242.1) and base fragment (m/z near 126.1) highlighted by arrows from samples also match standard 5mC. (E, F, G, H) Quantification of Met, SAM, and 5mC levels in *M*. *oryzae* mycelium samples. The 5mC concentrations are listed as 5mC per billion dC. Error bars represent the SD and different letters indicate significant differences (*P* < 0.05) tested by one-way ANOVA with Duncan’s post hoc test for (E and F) and significant differences were tested by Student’s t test for (G and H) (*P < 0.05).

Since HOA is the key enzyme required for Met biosynthesis, we used UPLC-ESI-MS/MS to analyze Met and SAM levels in WT, Δ*Momet2*, Δ*Mocys2*, and ΔΔ*Mocys2Momet2*. A SAM peak matching the retention time (0.37 minutes) of the SAM standard was detected in mycelium samples (Fig 3A). The Δ*Momet2*, Δ*Mocys2*, and ΔΔ*Mocys2Momet2* mutants had significantly lower levels of Met and SAM than WT (Figs 3E, 3F and S7). To test whether MoMet2 or MoCys2 affect DNA methylation, we examined 5mC levels in these strains by UPLC-ESI-MS/MS. A peak matching the retention time of the 5mC standard was found in the DNA of *M*. *oryzae* (Fig 3C). Moreover, the same base fragment was detected in two samples by MS/MS of 242.1 (mass/charge ratio), which also matched the 5mC standard (Fig 3D). We estimated 5mC abundance and found the global methylation levels (mC/total C) in the Δ*Momet2* mutant was significantly higher than that in WT (Fig 3G). No changes were found in the Δ*Mocys2* mutant (Fig 3H). These results indicated that disruption of the *MoMET2* gene alters global methylation in *M*. *oryzae*.

### The Δ*Momet2* mutant has a higher level of 5mC at the gene body and the promoter

**Fig 4:**
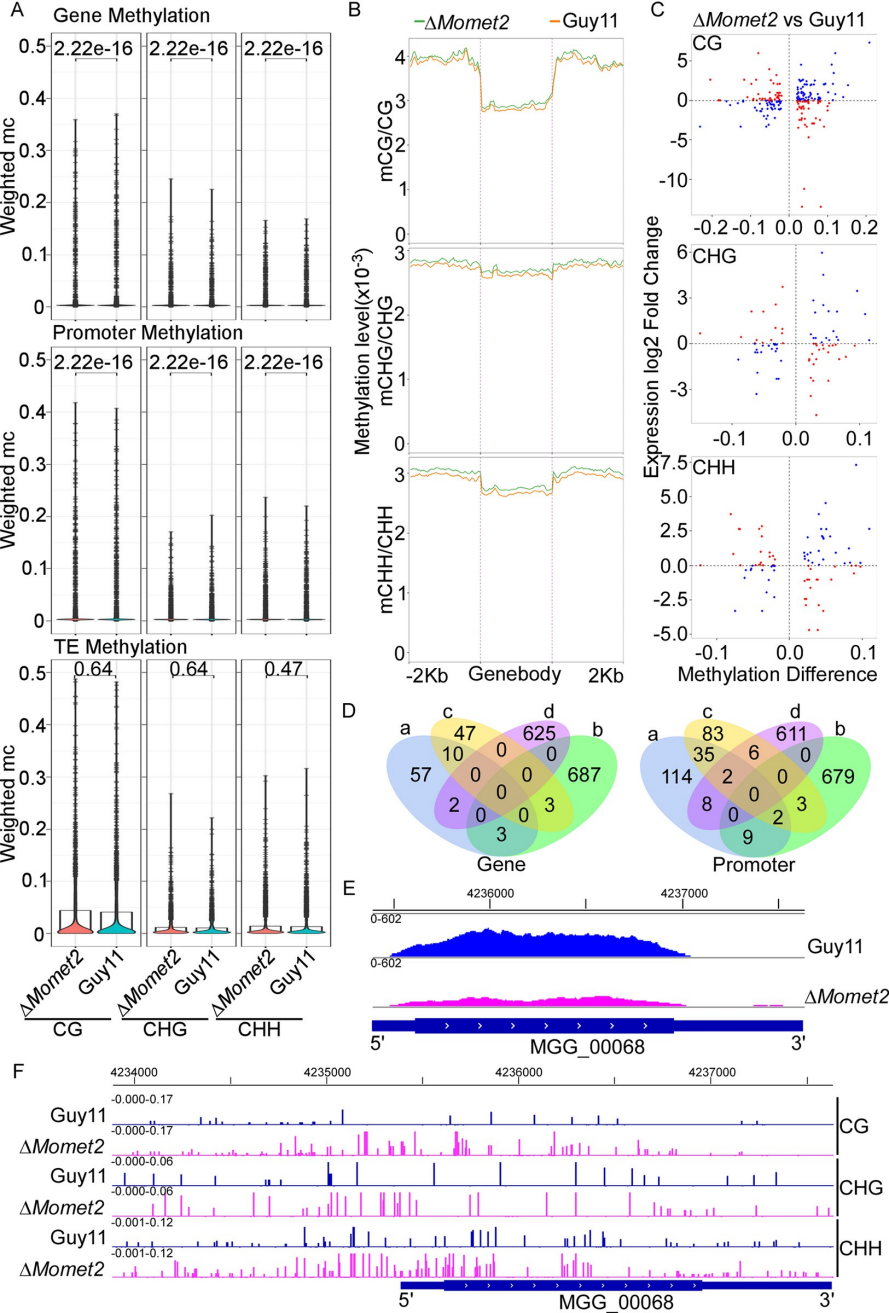
Integrative analysis of WGBS and RNA-seq identified transcripts affected by *MoMET2*. (A) Violin plot of the distribution of DNA methylation levels quantified as weighted methylation over genes, promoters, and transposable elements (TEs). Cytosine methylation was analyzed in the CG, CHG, and CHH sequence context. Methylation was measured in WT and Δ*Momet2* mutant strains. By averaging the weighted values of comparable methylation in the same original of the repeated samples, the Wilcox test-based p values for the number on the figures were determined. *P* < 0.05 represent significant differences. (B) 5mC profiles in gene regions. The 2 kb upstream and downstream flanking coding regions were aligned for all genes. TSS, transcription start site; TES, transcription end site. (C) Plot showing integrative analysis of WGBS and RNA-seq to analyze derepressed and repressed transcripts containing significantly altered 5mC modification. The genes associated with hypomethylation and high expression and genes associated with hypermethylation and low expression, red dots. The genes associated with hypomethylation and low expression and genes associated with hypermethylation and high expression, blue dots. (D) Venn diagram showing the overlaps between expressed genes and 5mC-marked genes. a, DMR Hypergenes, b, DEG upgenes, c, DMR Hypogenes, d, DEG downgenes. (E, F) The DNA methylation levels surrounding promoters and gene body regions, and the relative gene expression levels, were shown with screenshots of the Integrative Genomics Viewer (IGV) display. Guy11, blue. Δ*Momet2*, pink.

Since the Δ*Momet2* mutant had a significantly higher level of 5mC than WT, we hypothesized that gene disruption of *MoMET2* affects genome-wide DNA methylation patterns. To test this, we performed whole-genome bisulfite sequencing (WGBS) of mycelia from WT and Δ*Momet2* (two biological replicates) and found that the overall DNA methylation level was low, with an average weighted methylation percentage (calculated as the number of reads supporting methylation over the number of cytosines sequenced) at CG dinucleotides of 1.14% (Table 1). In addition, DNA methylation in Δ*Momet2* differed in the gene body and promoter regions, but not TE (Fig 4A). 5mC profiling in methylated genes revealed that 5mC peaks tended to flank the transcriptional start site (TSS) with a clear depletion pattern near the TSS, especially in CG methylation sequences (Fig 4B).

**Table 1:** Summary of DNA methylation in *M*. *oryzae* wild-type (WT) and the Δ*Momet2* mutant as measured by whole genome bisulfite sequencing calculated over 10 kb non-overlapping windows.

Genotype	Avg. weighted mCG	Avg. weighted mCHG	Avg. weighted mCHH	Avg. fraction mCG	Avg. fraction mCHG	Avg. fraction mCHH
Δ*Momet2*	0.0114	0.0048	0.0065	0.0330	0.0076	0.0136
Guy11	0.0107	0.0046	0.0062	0.0308	0.0071	0.0130

### MoMet2 regulates global gene expression in *M*. *oryzae*

To examine the potential effect of MoMet2 on gene expression regulation, we performed a transcriptome analysis involving WT and the Δ*Momet2* mutant (S8A Fig). Pearson correlation analysis revealed a distinct pattern of the global transcriptome between the two strains (S8B Fig). Additionally, differentially expressed gene (DEG) analysis (|fold change|>1.5) uncovered that 693 genes were significantly upregulated while 627 genes were downregulated in the Δ*Momet2* mutant (S8C Fig). The dendrogram suggested a high repeatability of WT and the Δ*Momet2* mutant WGBS data (S8D Fig). Most methylated cytosines were found to be localized in the repeat sequences (S8F Fig), consistent with the results of most previous studies [37]. Finally, we performed an integrative analysis of WGBS and RNA-seq on derepressed and repressed transcripts containing significantly altered 5mC modification (Fig 4C). The results showed that DNA methylation occurs in all sequence contexts: CG, CHG, and CHH (H = A, C, T), with CG methylation being predominant.

We next investigated the correlation between DNA methylation and gene expression in the Δ*Momet2* mutant. Among 170 genes with hypermethylated promoters in the Δ*Momet2* mutant, 11 were upregulated, and only 10 were downregulated. Furthermore, 2 genes were downregulated, and 3 genes were upregulated among 72 genes with hypermethylated gene bodies in the Δ*Momet2* mutant (Fig 4D). Of 170 genes with increased 5mC in promoters, only 10 were differentially expressed in the Δ*Momet2* mutant. Therefore, a conclusion that 5mC directly represses gene expression cannot be drawn.

Finally, to explore the biological processes MoMet2 might regulate, we performed a KEGG analysis on DEGs that were downregulated by 1.5-fold in the Δ*Momet2* mutant. The analysis revealed that the “biosynthesis of secondary metabolites” was highly enriched (S8E Fig). We therefore explored genes in this metabolic pathway with hypermethylation and low expression: MGG_00068 encoding a gliotoxin biosynthesis protein homolog, MoGliK. GliK has been demonstrated that plays critical roles in gliotoxin biosynthesis of *Aspergillus fumigatus* [38]. Indeed, compared to WT, the Δ*Momet2* mutant had significantly reduced MoGliK transcripts, accompanied by hypermethylation in the body and promoter region of the *MoGLIK* gene (Fig 4E and 4F).

### Altered virulence gene expression contributes to MoMet2-associated pathogenicity

**Fig 5:**
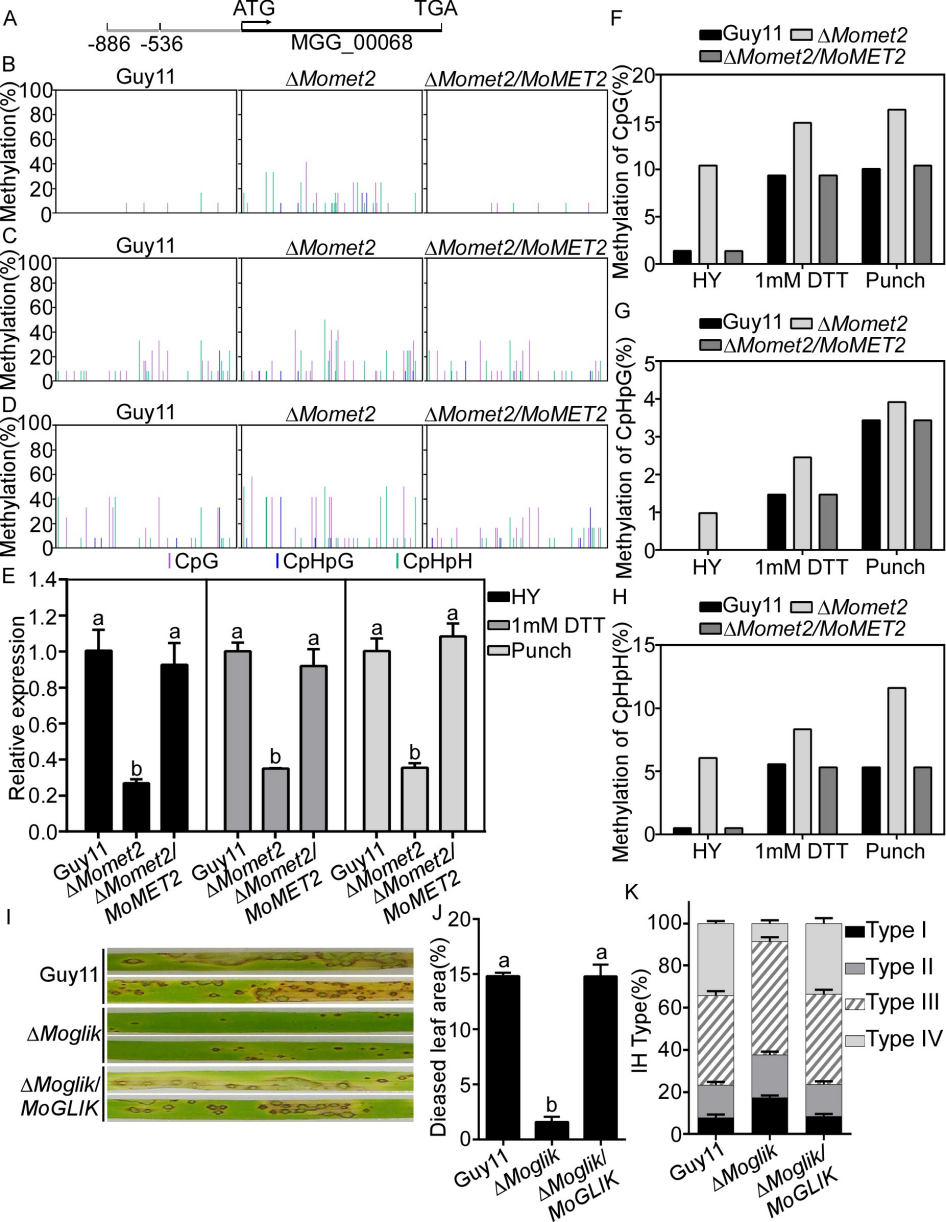
MoMet2 represses *MoGLIK* expression via DNA methylation on HY, stress, and infection. (A, B, C, D) Distribution of cytosine DNA methylation in three contexts in 350 bp of the *MoGLIK* promoter region in the Guy11, Δ*Momet2* and the complemented mutant Δ*MoMET2*/*MoMET2* HY. (B) HY added 1mM DTT (C) and punched rice (D) as measured by bisulfite sequencing. Sequencing data were analyzed using Kismeth software. CpG, lilac; CpHpG, blue; CpHpHp, green. At least 12 clones were sequenced per sample. (E) The relative expression of *MoGLIK* in different treatments. (F, G, H) Methylation levels of CpG, CpHpG, and CpHpHp in Guy11, Δ*Momet2* and the complemented mutant Δ*MoMET2*/*MoMET2*. (I) Pathogenicity test on rice leaves. (J) Diseased leaf area analysis. Data are presented as a bar chart showing the percentage of lesion area analyzed by ImageJ. (K) Statistical analysis for each type of IH shape. Error bars represent the SD and different letters indicate significant differences (*P* < 0.05) tested by one-way ANOVA with Duncan’s post hoc test for (E and J).

In plants, cytosine methylation primarily occurs in CG, CHG, and CHH (H = A, T, or C). We performed a bisulfite conversion reaction using a sequence starting 350 bp upstream of the transcription start site of *MoGLIK* to analyze the methylation status in its promoter region (Fig 5A). Consistent with the omics data, the CG, CHG, and CHH methylation levels were higher in the Δ*Momet2* mutant than in WT and complementation strains at the mycelium stage (Figs 5B, 5F, 5G, 5H and S10A), accompanied by lowered *MoGLIK* transcripts in the Δ*Momet2* mutant (Fig 5E). To examine the methylation status in response to external stress or during infection, we calculated the methylation levels of WT, Δ*Momet2* mutant, and complementation strains under 1 mM DTT stress treatment or after punch inoculation. The CG, CHG, and CHH methylation levels in the promoter region of *MoGLIK* in the Δ*Momet2* mutant were higher than that in WT and complementation strains after DTT stress treatment (Figs 5C, 5F, 5G, 5H and S10B) or punch inoculation (Figs 5D, 5F, 5G, 5H and S10C), accompanied by lowered *MoGLIK* transcripts in the Δ*Momet2* mutant (Fig 5E).

To examine the role of MoGliK in pathogenicity, we inoculated conidial suspensions of WT, the Δ*Moglik* mutant, and the complemented strains on the susceptible rice cultivar CO39. The Δ*Moglik* mutant displayed significantly reduced virulence while the complemented strain was similar to WT (Fig 5I and 5J). In the host penetration assay, the Δ*Moglik* mutant showed 17% type I, 20% type II, 54% type III, and 9% type IV IH growth while WT and the complemented strains had 8% type I, 16% type II, 42% type III, and 34% type IV IH growth (Fig 5K). These results indicated that MoGliK is required for pathogenicity and IH growth.

Similar results were also observed for MGG_07949 encoding a protein MoCDH-cyt with cytochrome domain of cellobiose dehydrogenase which is an extracellular hemoflavoenzyme secreted by fungi to assist lignocellulolytic enzymes in biomass degradation [39], which was randomly selected in the united analysis with hypermethylation and low expression (S9 Fig). Bisulfite sequencing analysis of methylated cytosine at *MoCDH*-*CYT* promoter showed that CG, CHG, and CHH methylation levels were higher in the Δ*Momet2* mutant than that in WT at mycelium growth stages and during punch inoculation (S9B, S9C, S9F, S9G, S9H and S11 Figs), accompanied by lower *MoCDH*-*CYT* transcripts in the Δ*Momet2* mutant (S9D Fig). Moreover, the Δ*Mocdh*-*cyt* mutant displayed a significantly reduced virulence in comparison to the WT and complemented strains (S9E Fig).

Taken together, these results indicated that MoMet2 regulates gene expression via 5mC methylation.

### Host-induced gene silencing (HIGS) in *MoMET2* and *MoCYS2* improves rice blast resistance

**Fig 6:**
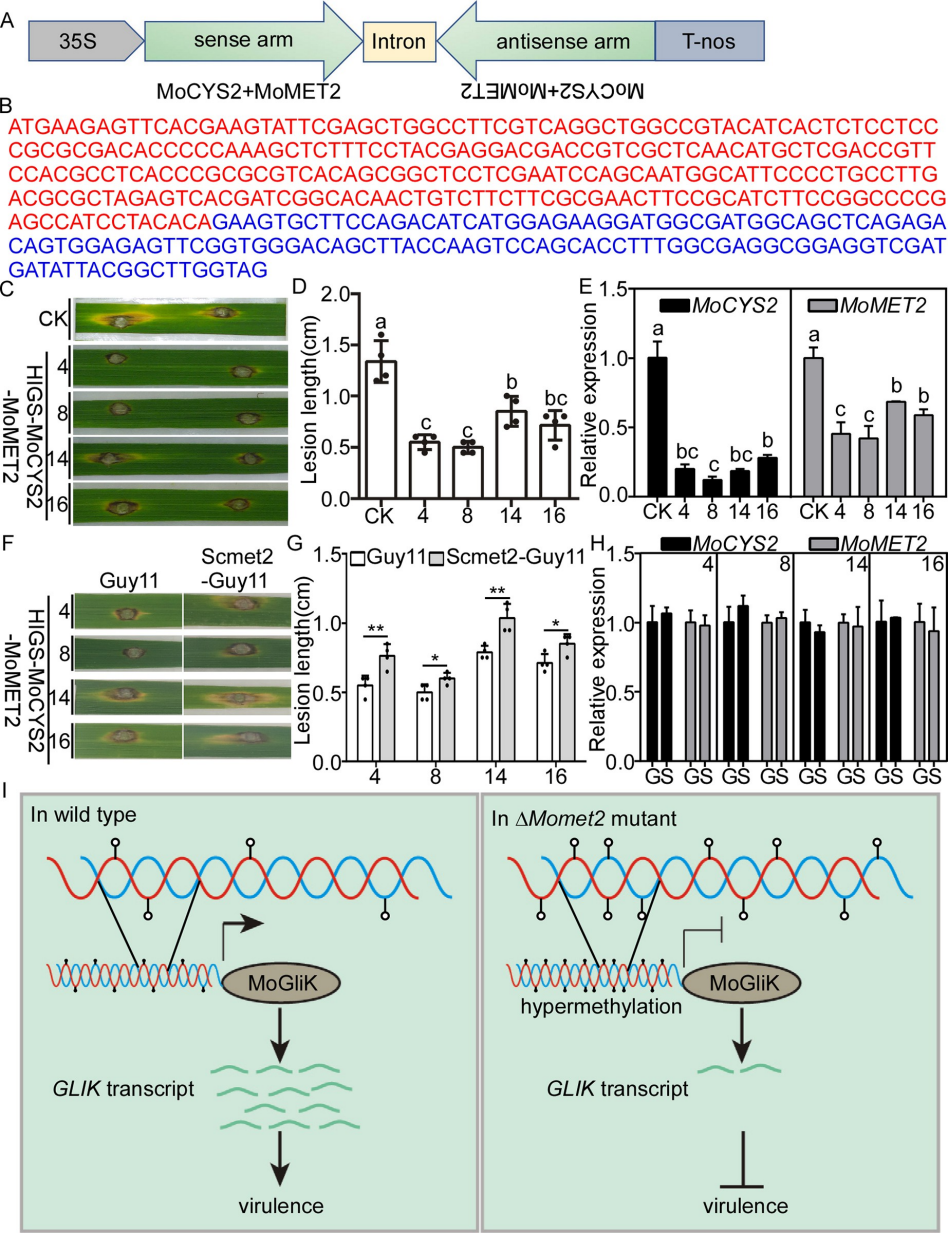
HIGS of chimeric *Mo*-*CYS2*-*MET2* results in strong resistance to rice leaf. (A) Schematic map of the *Mo*-*CYS2*-*MET2* RNAi cassettes. TrpC, fungus promoter; TtrpC, fungus terminator; 35S, plant promoter; NOS, plant terminator. (B) Sequence representation of *Mo*-*CYS2*-*MET2* RNAi fragments. *MoCYS2* and *MoMET2* are indicated in red and blue, respectively. (C) Disease phenotypes of CK and HIGS-*MoCYS2*-*MET2* transgenic plants at 7 dpi by *M*. *oryzae* Guy11. Rice cultivar CK and HIGS-*MoCYS2*-*MET2* were punch-inoculated with Guy11 (1 × 10^5^ spores ml^-1^). CK, the rice cultivar Zh11 expressing empty vector. (D) Quantitative analysis on lesion length of rice after infection by Guy11 at 7 dpi. (E) Relative mRNA expression of two target genes of *M*. *oryzae* during Guy11 infection of transgenic lines and CK at 7 dpi. (F) Disease phenotypes of CK and HIGS-*MoCYS2*-*MET2* transgenic plants at 7 dpi by the strain expression the *S*. *cerevisiae* ortholog *in M*. *oryzae* Guy11 (Scmet2-Guy11). Rice cultivar CK and HIGS-*MoCYS2*-*MET2* were punch-inoculated with Scmet2-Guy11 (1 × 10^5^ spores ml^-1^). CK, the rice cultivar Zh11 expressing empty vector. (G) Quantitative analysis on lesion length of rice after infection by and Guy11 and Scmet2-Guy11 at 7 dpi. (H) Relative mRNA expression of two target genes of *M*. *oryzae* during Scmet2-Guy11 and Guy11 infection of transgenic lines at 7 dpi. G, transgenic lines inoculating with Guy11; S, transgenic lines inoculating with Scmet2-Guy11. (I) Proposed model for the roles of methylation of the *MoGLIK* promoter in regulating pathogenicity. Error bars represent the SD and different letters indicate significant differences (*P* < 0.05) tested by one-way ANOVA with Duncan’s post hoc test for (D and E). Significant differences were tested by Student’s t test for (G and H): *P < 0.05, **P < 0.01.

HIGS is a powerful strategy for developing transgenic rice cultivars to control fungal diseases. Since MoMet2 and MoCys2 contribute to *M*. *oryzae* pathogenesis and there were no *MoMET2* and *MoCYS2* homologs in rice (Fig 1), they became the ideal targets for HIGS. After removing the conserved sequence, a 264-bp and a 129-bp DNA sequence remained in the MoCys2- and MoMet2-coding regions, respectively. To design a chimeric hairpin RNAi construct that could silence every single gene or simultaneously two target genes (Fig 6A), the selected fragments (Fig 6B) were used to generate a dsRNA sequence with a hairpin structure. The chimeric RNAi construct was first transformed into the WT strain, and the correspondingly silenced target genes were analyzed (S12A, S13A and S14A Figs). The transformants exhibited phenotypes similar to deletional mutants in vegetative growth and virulence (S12B, S12C, S12D, S13B, S13C, S13D, S14B, S14C and S14D Figs). These results indicated that the selected sequence fragments effectively silence target genes in *M*. *oryzae*, and, thereby are suitable for HIGS.

Using the same DNA sequence, we generated a siRNA construct and introduced it into Zhonghua11 (Zh11) for HIGS (HIGS-*MoCYS2*-*MET2*). We also generated the rice cultivar Zh11 expressing the empty vector as a negative control (CK). We then inoculated Guy11 spore suspensions onto CK and HIGS-*MoCYS2*-*MET2* transgenic plants. At 7d post-inoculation (dpi), the HIGS-*MoCYS2*-*MET2* plants produced significantly smaller lesions than CK (Fig 6C and 6D). To verify whether resistance to *M*. *oryzae* in the transgenic rice lines was caused by in planta-derived silencing of the two target genes, we quantified the transcript levels of *MoMET2* and *MoCYS2* during Guy11 infection of leaves from T1 transgenic and CK at 7 dpi. The qPCR analysis showed that the transcripts of *MoMET2* and *MoCYS2* were reduced by approximately 30%-55% and 72%-88%, respectively, in four transgenic lines, compared to CK (Fig 6E). We further inoculated HIGS-*MoCYS2*-*MET2* transgenic plants with Guy11 and expressing the Scmet2 in Guy11 (Scmet2-Guy11), respectively. At 7 dpi, the HIGS-Mo*CYS2*-*MET2* plants infected with Guy11 produced significantly smaller lesions than that infected with Scmet2-Guy11 (Fig 6F and 6G). Moreover, there was no significant difference in the transcripts of *MoMET2* and *MoCYS2* (Fig 6H). These findings indicated that HIGS-*MoCYS2*-*MET2* improves rice resistance against *M*. *oryzae*.

## Discussion

Although available antifungal agents are effective in combating rice blasts, the emergence of resistance to these agents facilitates a new understanding of fungal pathogens, plant-fungi interactions, and virulence factors. The study of new antifungal drug targets and a better understanding of fungal pathogenesis are both anticipated benefits of characterizing virulence factors. In our study, we identified and characterized the functions of MoMet2 and MoCys2 from *M*. *oryzae*. We found that MoMet2 regulates gene expression via 5mC modification during infection and that *MoGLIK* is one of the key target genes (Fig 6I). In WT, hypomethylation of the *MoGLIK* promoter and gene body leads to its high transcription, which contributes to *M*. *oryzae* virulence. In contrast, the *MoGLIK* promoter and gene body are highly methylated in the Δ*Momet2* mutant, which impedes *MoGLIK* transcription to inhibit the pathogenicity of *M*. *oryzae*. Of note, HIGS targeting *MoMET2* and *MoCYS2* effectively controls rice blasts. Our studies revealed the molecular mechanisms of *MoMET2* mediating gene expression via 5mC modification in *M*. *oryzae* and provided a candidate target that can be used as both a novel fungicide target and in HIGS to effectively control the rice blast.

In the last few years, the stable plant transformation-based HIGS system has been proven to efficiently control certain pests, nematodes, filamentous pathogens, and parasitic plants [40–44]. Target gene selection is the most important prerequisite for HIGS. In general, genes that play a vital role in pathogen growth, development, and pathogenicity are considered HIGS target candidates. However, the selected gene fragment, size, and sequence specificity, as well as positions of dsRNA, all need to be considered for HIGS, and thus few effective HIGS targets have been uncovered [45–47]. Met biosynthetic pathways are often focused because they are absent from mammals, making biosynthetic enzymes attractive targets for antimicrobial discoveries. Met is widely involved in vegetative growth, asexual development, multiple stress resistance, and the pathogenicity of various fungal pathogens. HOA participates in the first committed step of Met metabolism, and loss of HOA function causes defects in fungal development, and virulence [33]. In *Cryptococcus neoformans*, a small-molecule screen identified the first inhibitor of fungal HOA [31]. In *Candida albicans*, L- and D-penicillamine exhibit antifungal activities by inhibiting the function of HOA [32]. These findings provide support for the ability to target HOAs with antifungal compounds. Consistent with these findings, transgenic rice produced in our HIGS experiments exhibited strong resistance to *M*. *oryzae* (Fig 6C–6E) due to the downregulated expression of *MoCYS2* and *MoMET2* by HIGS (Fig 6F–6H). It would be interesting to test whether existing HOA inhibitors are effective against *M*. *oryzae*, and to develop novel fungicides with stronger effects targeting HOA.

Met is not only an essential amino acid for various biological processes but also can be converted to SAM, donating methyl groups to more than 80 biological reactions [48]. Some Met-related genes have been previously reported to be associated with the pathogenicity of *M*. *oryzae* [49–52], but the relationship between them and DNA methylation is unclear. A positive correlation between SAM contents and DNA methylation has been hypothesized and confirmed in some studies [53]. However, a previous study suggested that the SAM content of female mice liver was reduced by methyl deficiency while their 5mC content was rather increased [54]. Additionally, 12 h cultures of Met-starved *met-1* mutant and Cys-starved *cys-3* mutant in *Neurospora crassa* displayed ~3-fold less SAM than did WT cultures, whereas their methylation levels appeared comparable with WT [55]. These suggested that SAM contents is not always linear to DNA methylation in the genome. Subtle reduced SAM would have drastic cellular effects including induced DNA methylation, when SAM is reduced to a certain level, SAM primarily provides methyl groups for the DNA methylation reaction [56], maybe a reason of resulting in significantly increased DNA methylation in the Δ*Momet2* mutant (Fig 3G). The Δ*Mocys2* mutant showed no effects on the methylation (Fig 3H), indicating that other methyl donors [57] may substitute SAM for its function on DNA methylation in fungi. Moreover, the DNA methylation process showed its complexity among organisms, even in the case of methyltransferase knockout, not all changes occur in a singular direction [58].

Not only that, the fluctuation of Met content alter the methylation levels of mammalian including some locus-specific DNA and histone methylation, which is sensitive to Met [59, 60].It has been demonstrated that hypermethylation in the reelin promote in the cortex of mice upon Met supplementation [61] and administration of Met led to prominent hypermethylation of the brain-derived neurotrophic factor (Bdnf) DNA [62]. The methylation of *MoGLIK* was similar to Guy11 in the complemented strains (Figs 5 and S10), indicating that Met2 is associated with *MoGLIK* methylation in direct or indirect manner. Based on these results, we considered that maybe MoGliK was sensitive to DNA methylation which leads to the only gene was evaluated among this gli gene cluster.

DNA methylation links genetic components and environmental changes. The global DNA methylation level is very low in fungi when compared to other eukaryotes [63]. However, it plays an important role in gene regulation and development [64]. In *M*. *oryzae*, the existence of DNA methylation was examined by restriction enzyme-based work on a retrotransposon, *MAGGY* [19]. A comparative methylome analysis showed that mycelia are the tissue with the highest number of methylated cytosine and that significantly proportion of these methylation sites disappears in conidia and appressoria of *M*. *oryzae* [18], suggesting that DNA methylation patterns change according to developmental stage. In contrast, our studies revealed a difference in DNA methylation in the promoter and gene body between WT and the Δ*Momet2* mutant especially during infection. Our findings thus highlight the importance of virulence gene expression regulated by DNA methylation in *M*. *oryzae*.

Finally, during the characterization of genome-wide methylation in *M*. *oryzae*, we attempted to inject fungi spores into the bundle sheath cells and collected IH by microdissection. However, the bundle sheath cells were too thin to be fixed, and the amount of IH was insufficient for sequencing analysis, despite separating bundle sheath-infected cells by single-cell flow cytometer. In resequencing to examine the ratio of plant genome to pathogen genome during infection, *M*. *oryzae* accounted for only 1.044% of the detected genomes, with the rest belonging to the plant (S15 Fig). Therefore, it is difficult to distinguish fungi genome-wide DNA methylation during plant-fungi interactions. To circumvent the difficulty in distinguishing fungal DNA methylation during infection, we examined the role of methylation during stress and infection at the individual level. Stress treatment with DTT (1 mM) and punching resulted in the same trend as the mycelial stage, whereby the DNA methylation level of target genes is higher in the Δ*Momet2* mutant than in WT. Comparison of the dynamic of DNA methylation under different development and infection stages may suggest the critical role of DNA methylation in altering pathogen strategies to infect its host plants. Our studies revealed differences in methylation levels from promoter and gene body regions at the mycelial stage, and this difference is most drastic during infection. Therefore, monitoring the fungus genome-wide DNA methylation status in the plant-fungi interaction with deficient methylation levels is expected to develop more accurate or targeted methods.

## Materials and methods

### Phylogenetic tree analysis and yeast complementation assays

To construct the phylogenomic tree, we chose numerous common grass and their pathogenic fungi, including *Oryza sativa*, *Sorghum sativa*, *Triticum aestivum*, *Zea mays*, *Tilletia caries*, *Rhizoctonia solani*, *Puccinia sorghi*, *Puccinia striiformis*, *Sporisorium reilianum*, *Ustilago maydis*, *Blumeria graminis*, *Colletotrichum graminicola*, *M*. *oryzae*, *Fusarium graminearum*, *Fusarium verticillioides*, *Claviceps purpurea*, *Ustilaginoidea virens*, *Macrophomina phaseolina*, *Cercospora zeae-maydis*, *Curvularia lunata*, *Pyrenophora tritici-repentis*, *Bipolaris maydis*, *Bipolaris sorokiniana* and *Bipolaris oryzae*. In total, 469 unique encoded proteins shared by all genomes were filtered using orthofinder, and subsequently aligned with mafft (mafft-linsi-anysymbol). The phylogenomic tree was constructed using FastTree based on the alignments of single-copy ortholog families with approximately-maximum-likelihood model and bootstrap 1000. Proteins with high query coverage to MoMet2 were sourced from the NCBI database website (https://www.ncbi.nlm.nih.gov/). Amino acid sequence alignment was performed with CLUSTALW and resulting alignment was further processed to construct the phylogenetic tree using MEGA 7.0.

To test the homologies of *MoMET2* and *MoCYS2* to *ScMET2*, the full-length cDNA of *MoMET2* and *MoCYS2* were individually cloned into the vector pYES2 (Invitrogen). Subsequent to PCR sequencing and screening on SD medium without uracil, the MoMet2-pYES2, MoCys2-pYES2 constructs and MoMet2-pYES2, along with MoCys2-pYES2 combined constructs, were each transformed into the yeast Δ*Scmet2* mutant (BY4741 loss of YNL277W). Yeast colonies were cultured on YPD medium (10 g Yeast Extract, 20 g Peptone, 20 g D-Glucose) and adjusted to an OD600 value of 0.1. Following this, 10-fold serial dilutions were cultured on SD-Met (galactose) plates at 30°C for 4 d and subsequently [65].

### Strains and cultural conditions

The wild type (WT) was Guy11 strain in this study. For vegetative growth, small agar blocks (2 × 2 mm) were cut from the edge of 4-d-old cultures and placed onto complete medium (CM), minimal medium (MM) or straw decoction and corn (SDC) for culture in the dark at 28°C for 7 d. The diameter growth was measured after incubation for 7 d. Liquid CM medium was used to prepare mycelia for DNA and RNA extraction. For conidia production, mycelia were grown in the dark on SDC or CM medium at 28°C for 7 d, followed by constant illumination for 3 d under fluorescent light [66].

### Targeted gene deletion and complementation

The gene deletion mutants were generated using the standard one-step gene replacement strategy. First, two 1.0 kb sequences flanking the targeted genes were PCR amplified with primer pairs (S1 Table). Then, the resulting PCR products of *MoMET2* were digested with restriction endonucleases and ligated with the *HPH* cassette released from pCX62. Finally, the completed inserts were sequenced. The 3.4 kb fragments, which contain the flanking sequences and hygromycin resistance cassette, were amplified and transformed into protoplasts of WT. Putative mutants were screened by PCR and confirmed by Southern blotting analysis. The complement fragments, which contain the entire *MoMET2* gene with its native promoter regions, were amplified by PCR (Phanta Super-Fidelity DNA Polymerase, Vazyme Biotech Co., Nanjing, China) with primers (S1 Table) and inserted into pYF11 (bleomycin resistance) to complement the respective mutant strains. Other gene deletion mutants and corresponding complemented strains were obtained using the same strategy.

### Virulence and invasive growth assays

For infection, conidia were harvested from 10-d-old SDC agar cultures, filtered, and resuspended to a concentration of 5 × 10^4^ spores ml^-1^ in a 0.2% (w/v) gelatin solution. The conidia were sprayed onto two-week-old seedlings of rice (*Oryza sativa* cv. CO39) [67]. For rice leaves, 5 ml of a conidial suspension of each treatment was sprayed. For the detached barley inoculation assay, conidia were suspended to a concentration of 1 × 10^5^ spores ml^-1^ in a 0.2% (w/v) gelatin solution, and 20 μl conidia were inoculated onto leaves of 7-day-old barley seedlings [68]. Inoculated plants were kept in a growth chamber at 25°C with 90% humidity and in the dark for the first 24 h, followed by a 12 h /12 h light /dark cycle. Lesion formation was observed daily and recorded by photography 7 dpi. The degree of disease lesions was analyzed by ImageJ [69].

For observation of the penetration and invasive growth in rice cells, conidial suspensions (1 × 10^5^ spores ml^-1^) of WT, mutants and complement strains were injected into rice leaf sheath. The inner epidermises of infected sheaths were harvested at different hours post-inoculated by WT and mutant strains and observed under a microscope.

### UPLC-ESI-MS/MS analysis for Met, SAM, and 5mC

A HPLC-ESI-MS/MS system, consisting of electrospray-time-of-flight mass spectrometry (Triple TOF 5600+, AB Sciex) and liquid chromatography (LC-20ADXR HPLC, Shimadzu), was used for 5mC detection. Data acquisition and processing were performed using PeakView version 2.0 (AB Sciex). The HPLC separation was performed on a reversed-phase column (C18, 2.1 mm × 100 mm, 2.6 μm; Kinetex) with a flow rate of 0.2 ml/min at 40°C. Formic acid (FA) in water (0.1%, v/v, solvent A) and FA in methanol (0.1%, v/v, solvent B) were employed as the mobile phase. A gradient of 0.5 min 2% B, 2 min 10% B, 3 min 90% B, 3.5 min 2% B and 5 min 2% B was used. The mass spectrometry detection was performed under ESI positive mode with a DuoSpray dualion source. The retention time of 5mC is around 0.74 min. The mass transition for 5mC was set as m/z 242.11 > 126.1 [70].

Su et al. developed a UHPLC-ESI-MS/MS method to simultaneously determine the levels of 14 metabolites, including 4 Met metabolism metabolites (Met, homocysteine, SAM and S-adenosylhomocysteine) [71]. Based on the sample preparation and experimental methods, we discovered the ideal test suitable for *M*. *oryzae*. The ACQUITY UPLC BEH Amide column (2.1 × 100 mm, 1.7 μm, Waters, USA) was used for metabolite separation at 30°C. The mobile phases were water (A) and Acetonitrile (ACN) (B) both containing 0.1% FA. The LC gradient program was optimized as follows: 0.5 min 15% B, 3 min 85% B, 4 min 85% B, 4.01 min 15% B, 5 min 15% B. The injection volume was 1.0 μl.

### RNA extraction, RNA-seq, and qRT-PCR

Total RNA as isolated from 2-d-old hyphae using Omega Total RNA Kit I according to the manufacturer’s manual. RNA quantity and quality was measured using Nanodrop ND-1000 and 1% agarose gel electrophoresis. RNA-seq service was provided by Novogene Corporation (Beijing, China). First-strand of cDNA was synthesized from 1 μg total RNA using HiScript III RT SuperMix (R323, Vazyme, China). ChamQ Universal SYBR qPCR Master Mix (Q711, Vazyme, China) was used for quantitative RT-PCR (qRT-PCR) reactions. QRT-PCR was performed using the ABIPRISM 7500 Fast Real-Time PCR System. The expression of each gene was normalized to the expression of internal control ACTIN, and primers used for qRT-PCR were listed in S1 Table.

### Whole-Genome bisulfite sequencing and analysis

Genomic DNA of 2-d-old Guy11 and the Δ*Μοmet2* mutant mycelium were isolated using TIANGEN DNAquick Plant Systerm (DP321) according to the manufacturer’s manual. Genomic DNA degradation and contamination was validated by agarose gels. DNA purity was checked using NanoPhotometer spectrophotometer (IMPLEN, CA, USA). DNA concentration was measured using Qubit DNA Assay Kit in Qubit 2.0 Flurometer (Life Technologies, CA, USA).

A total amount of 100 ng genomic DNA spiked with 0.5 ng lambda DNA was fragmented by sonication to 200–300 bp with Covaris S220. These DNA fragments were treated with bisulfite using EZ DNA Methylation-Gold Kit (Zymo Research), and the library was constructed by Novogene Corporation (Beijing, China). Subsequently, pair-end sequencing of the sample was performed on Illumina platform (Illumina, CA, USA). Library quality was assessed on the Agilent Bioanalyzer 2100 system.

The library was sequenced on Illumina Novaseq platform. Image analysis and base calling were performed with Illumina CASAVA pipeline, and finally generated 150bp paired-end reads.

We used FastQC (fastqc_v0.11.5) to perform basic statistics on the quality of the raw reads. Then, those read sequences produced by the Illumina pipeline in FASTQ format were pre-processed through fastp (fastp 0.20.0). The remaining reads that passed all the filtering steps was counted as clean reads, and all subsequent analyses were based on this. Finally, we used FastQC to perform basic statistics on the quality of the clean data reads.

### Bisulfite sequencing

Genomic DNA was extracted from WT and the Δ*Μοmet2* mutant mycelium. Bisulfite treatment was conducted using the EZ DNA Methylation-Gold Kit (D5005, Zymo Research, USA). Based on MGG_00068, MGG_07949 promoter sequences, the bisulfite primers were designed through Meth Primer software (http://www.urogene.org/cgi-bin/methprimer/methprimer.cgi) to test the methylation status of the target regions. The bisulfite-treated DNA was used for PCR amplification with ZymoTaq DNA Polymerase (E2001, Zymo Research, USA). The PCR products were cloned into the T-Vector pMD 19 (3271, TaKaRa, Japan) and 12 independent clones were sequenced for each variety. For each sample, at least 12 individual clones were sequenced, and the sequencing data were analyzed using Kismeth software (http://katahdin.mssm.edu/kismeth/revpage.pl) and CyMATE software (http://www.cymate.org/). The primer sequences used for bisulfite sequencing are listed in S1 Table.

### Construction of gene silencing vectors

The HIGS experiments mainly followed the method reported previously [72, 73]. The silencing vector pSilent-1 was constructed by inserting a PCR-amplified fragment of the *M*. *oryzae* CUT intron with multiple cloning sites. The 5’ multiple cloning site includes unique recognition sites for XhoI, SnaBI, and HindIII, and the 3’multi-cloning site includes those for BglII, SphI, StuI, KpnI, and ApaI [74]. We identified the protein’s conserved domains and thus presumed its putative function via the CD-Search interface (https://www.ncbi.nlm.nih.gov/Structure/cdd/docs/cdd_search.html). In order to silence the *MoCYS2* and *MoMET2* in *M*. *oryzae*, a 264-bp variable domain fragment of *MoCYS2* and a 129-bp variable domain fragment of *MoMET2* were synthesized, and were subsequently inserted into pSilent-1 in sense and antisense orientation, respectively. The constructed pSilent-1- *MoCYS2*, pSilent-1-*MoMET2* and pSilent-1-*MoCYS2*-*MoMET2* were co-transformed into the protoplasts of the WT strain. Then the fragment containing the gene-specific DNA fragments in two opposite orientations and the GUS linker were amplified and cloned into the pU2301 vector and the recombinant plasmids were used for rice transformation. Then the expression of target genes was analyzed by qRT-PCR.

### Statistical analysis

Results are presented as the mean ± standard deviation (SD) of at least three replicates. Student’s t tests were performed using Excel. One-way analysis of variation (ANOVA) followed by Duncan’s new-multiple range tests was performed using SPSS 2.0. Statistical tests are described in the figure legends.

## Supporting information

S1 Fig Amino acid sequence alignment. (A) Amino acid alignment of MGG_01469 and its ortholog met2 in *Saccharomyces cerevisiae*. (B) Amino acid alignment of MGG_14202 and its ortholog cys2 in *Colletotrichum chlorophyti*.
(TIF)

S2 Fig Phylogenetic and domain analysis of met2. (A) Amino acid alignment of MGG_01469 and its ortholog met2 in *Saccharomyces cerevisiae*. (B) Amino acid alignment of MGG_14202 and its ortholog cys2 in *Colletotrichum chlorophyti*.
(TIF)

S3 Fig Southern blot analysis of the related gene disruption mutants. (A) Amino acid alignment of MGG_01469 and its ortholog met2 in *Saccharomyces cerevisiae*. (B) Amino acid alignment of MGG_14202 and its ortholog cys2 in *Colletotrichum chlorophyti*.
(TIF)

S4 Fig Functional complementation of *S*. *cerevisiae* Δ*Scmet2* mutants. (A) Amino acid alignment of MGG_01469 and its ortholog met2 in *Saccharomyces cerevisiae*. (B) Amino acid alignment of MGG_14202 and its ortholog cys2 in *Colletotrichum chlorophyti*.
(TIF)

S5 Fig Functional complementation of *M*. *oryzae* Δ*Momet2* and Δ*Mocys2* mutants. (A) Vegetative growth of Guy11, Δ*Momet2*, Δ*Momet2*/*MoMET2*, Δ*Momet2*/*ScMET2* were grown on CM, MM, SDC. (B) Vegetative growth of Guy11, Δ*Mocys2*, Δ*Mocys2*/*MoCYS2*, Δ*Mocys2*/*ScMET2* were grown on CM, MM, SDC. Error bars represent SD and different letters indicate significant differences (*P* < 0.05) tested by one-way ANOVA with Duncan’s post hoc test.
(TIF)

S6 Fig Vegetative growth and virulence analysis recovery of mutants with Met or Cys supplementation. (A) Vegetative growth of the *M*. *oryzae* strains on CM MM and SDC supplemented with 1mM Met or 1mM Cys. (B) Conidia were observed under a light microscope after illumination for 24 h and photographed. (C, D) Statistical analysis of conidial numbers of the indicated strains. Error bars represent the SD and different letters indicate significant differences (*P* < 0.05) tested by one-way ANOVA with Duncan’s post hoc test. (E, F) Virulence analysis of the *M*. *oryzae* strains supplemented with 50 mM Met or 1 mM Cys.
(TIF)

S7 Fig Calibration curve correlating the peak area to the corresponding concentration of the test material. (A, B, C, D) Calibration curve of Met, SAM, dC and 5mC, respectively.
(TIF)

S8 Fig 5mC and RNA sequencing using mycelium of wild type and the Δ*Momet2* mutant. (A) Distribution of expression along the whole mRNA transcripts of *M*. *oryzae* detected in wild type strain and the *MET2* deletion mutant. (B) The heatmap showing similarities exanimated in correlation analysis between Guy11 and Δ*Momet2*. (C) Volcano plot of. Δ*Momet2* mutant gene expression pattern. (D) The dendrogram of sample clustering from Guy11 and Δ*Momet2* WGBS. (E) KEGG analysis of ∆*Momet2*—down genes from RNA sequencing. (F) Hyper/Hypo number distribution in DMR anchoring region.
(TIF)

S9 Fig Momet2 represses *MoCDH*-*CYT* expression via DNA methylation on HY and infection. (A, B, C) Distribution of cytosine DNA methylation in three contexts in 519 bp of the *MoCDH*-*CYT* promoter region in the Guy11 and Δ*Momet2* HY (B) and punched rice (C) as measured by bisulphite sequencing. Sequencing data were analyzed using Kismeth software. CpG, lilac; CpHpG, blue; CpHpHp, green. At least 12 clones were sequenced per sample. (D) The relative expression of *MoCDH*-*CYT* in different treatments. Error bars represent the SD (Student’s t test, *P < 0.05, **P < 0.01). (E) Pathogenicity test on rice leaves. (F, G, H) Methylation levels of CpG, CpHpG, and CpHpHp in the Guy11 and Δ*Momet2*.
(TIF)

S10 Fig Bisulfite sequencing analysis of methylated cytosine at the *MoGLIK* promoter. Bisulfite sequencing data of individual clones were submitted to the CyMATE program to analyze the methylated cytosines. Distribution of cytosine DNA methylation in three contexts in the *MoGLIK* promoter region in the Guy11, Δ*Momet2* and the complemented mutant Δ*MoMET2*/*MoMET2* HY (A) HY added 1 mM DTT (B) and punched rice (C) as measured by bisulfite sequencing.
(TIF)

S11 Fig Bisulphite sequencing analysis of methylated cytosine at the *MoCDH*-*CYT* promoter. Bisulfite sequencing data of individual clones were submitted to the CyMATE program to analyze the methylated cytosines. Distribution of cytosine DNA methylation in three contexts in the *MoCDH*-*CYT* promoter region in the Guy11 and Δ*Momet2* HY (A) and punched rice (B) as measured by bisulfite sequencing.
(TIF)

S12 Fig The *MoCYS2* strain was successfully silenced. (A) The relative expression of *MoCYS2* in different silenced strains. 2, 3, 5, 8, silenced strains. Error bars represent the SD and different letters indicate significant differences (*P* < 0.05) tested by one-way ANOVA with Duncan’s post hoc test. (B, D) Vegetative growth of the 8 silent stains on CM, MM, SDC. Error bars represent the SD and different letters indicate significant differences (*P* < 0.05) tested by one-way ANOVA with Duncan’s post hoc test. (C) Virulence analysis of the 8 silent stains on rice.
(TIF)

S13 Fig The *MoMET2* strain was successfully silenced. (A) The relative expression of *MoMET2* in different silenced strains. 1–10, silenced strains. Error bars represent the SD and different letters indicate significant differences (*P* < 0.05) tested by one-way ANOVA with Duncan’s post hoc test. (B, D) Vegetative growth of the 7 silent stains on CM, MM, SDC. Error bars represent SD and different letters indicate significant differences (*P* < 0.05) tested by one-way ANOVA with Duncan’s post hoc test. (C) Virulence analysis of the 7 silent stains on rice.
(TIF)

S14 Fig The *MoCYS2* and *MoMET2* were successfully silenced simultaneously. (A) The relative expression of *MoCYS2* and *MoMET2* in different silenced strains. 3–7, silenced strains. Error bars represent the SD and different letters indicate significant differences (*P* < 0.05) tested by one-way ANOVA with Duncan’s post hoc test. (B) Virulence analysis of the 7 silent mutants on barley. (C, D) Vegetative growth of the 7 silent stains on CM, MM, SDC. Error bars represent SD and different letters indicate significant differences (*P* < 0.05) tested by one-way ANOVA with Duncan’s post hoc test.
(TIF)

S15 Fig The ratio of fungi to rice in resequencing. (TIF)

S1 Table Primers used in this study. (XLSX)

S2 Table Comparison of mycological characters among Guy11 and deletion mutant strains. (XLSX)
