# Supplementary figures and images for: Methionine biosynthesis enzyme MoMet2 is required for rice blast fungus pathogenicity by promoting virulence gene expression via reducing 5mC modification
Source: PLoS Genet. 2023 Sep 21;19(9):e1010927. doi: 10.1371/journal.pgen.1010927 (PMC10547190; doi:10.1371/journal.pgen.1010927)

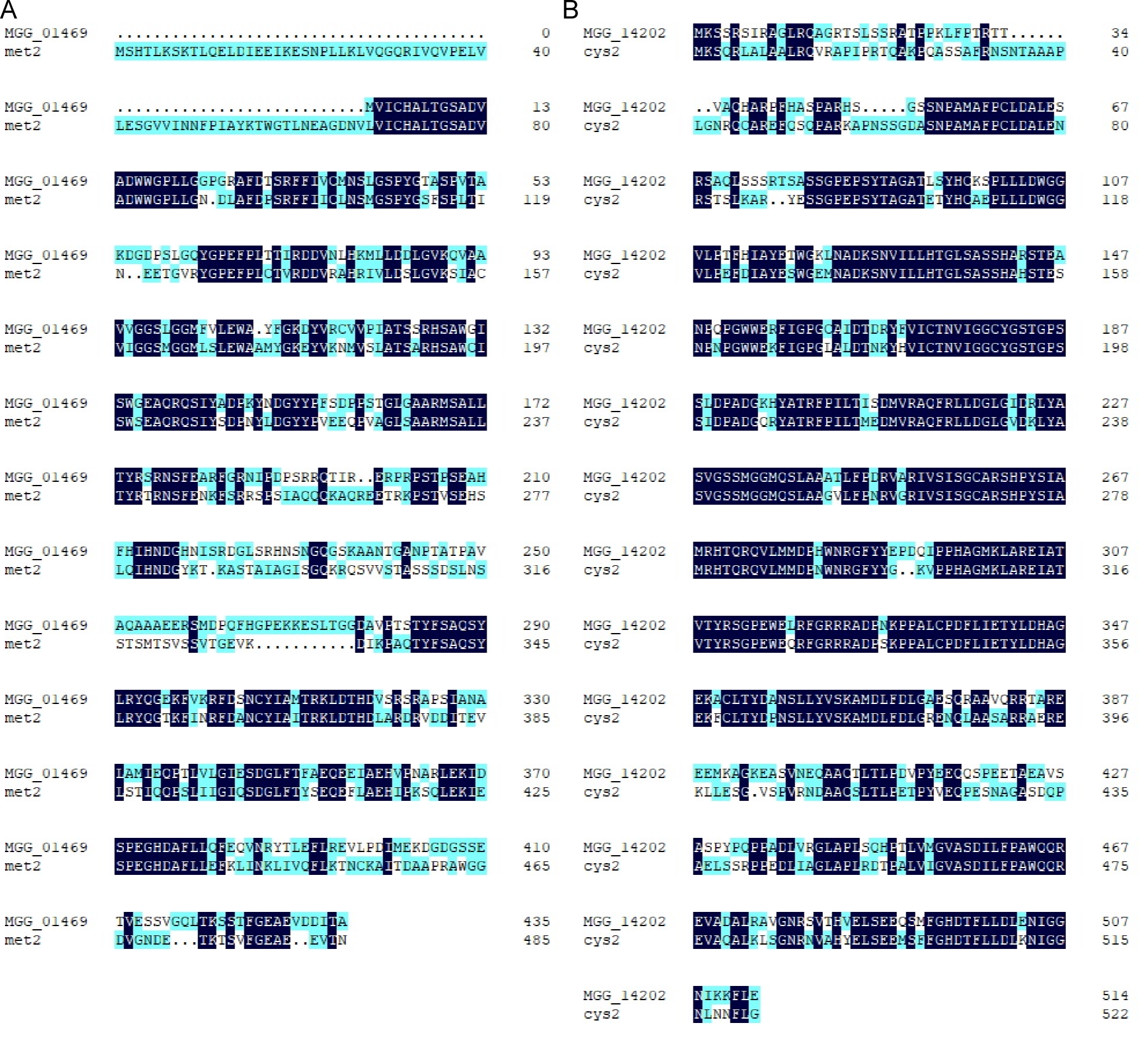

Supplement: S1 Fig — (A) Amino acid alignment of MGG_01469 and its ortholog met2 in Saccharomyces cerevisiae. (B) Amino acid alignment of MGG_14202 and its ortholog cys2 in Colletotrichum chlorophyti. (TIF) [file pgen.1010927.s001.tif]

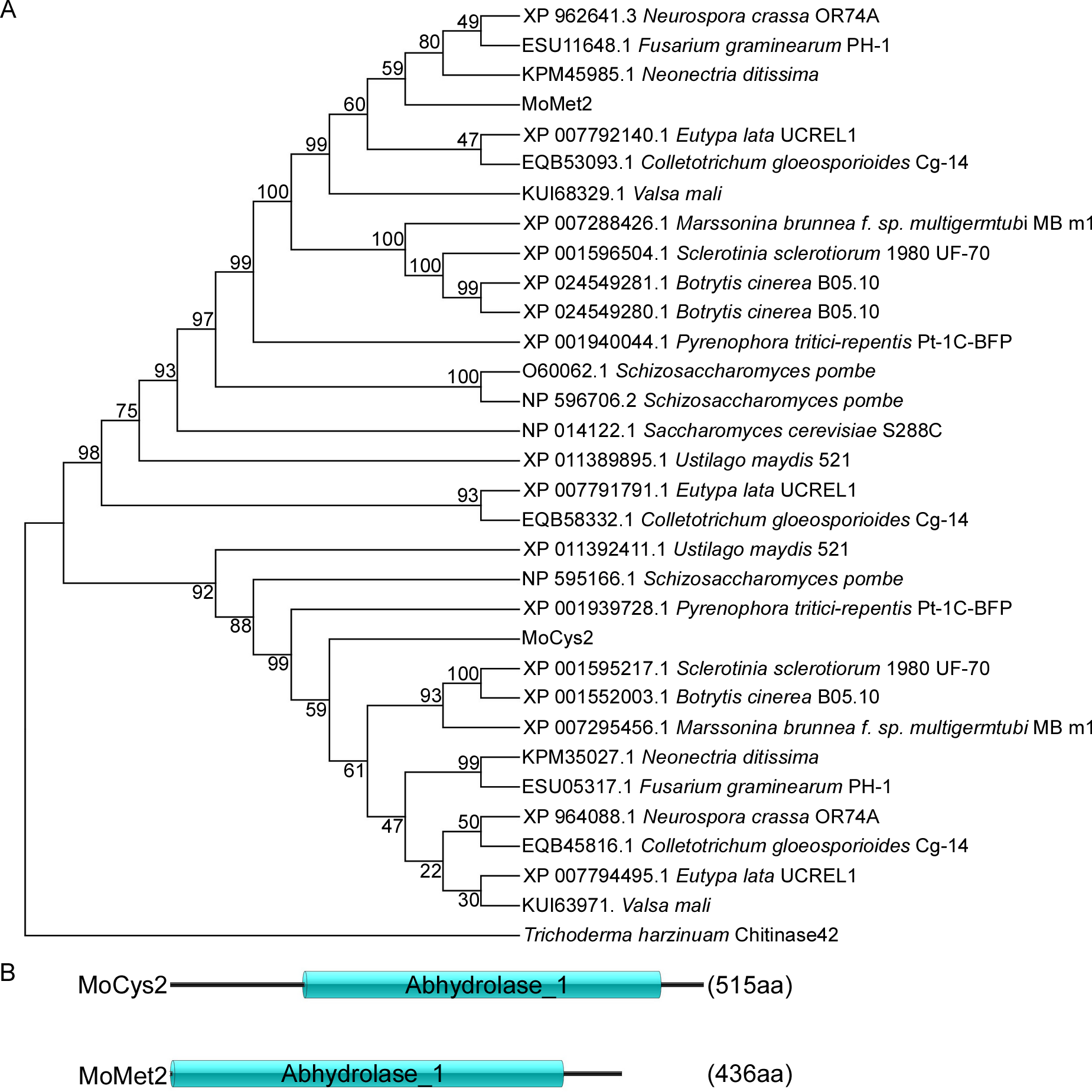

Supplement: S2 Fig — (A) Amino acid alignment of MGG_01469 and its ortholog met2 in Saccharomyces cerevisiae. (B) Amino acid alignment of MGG_14202 and its ortholog cys2 in Colletotrichum chlorophyti. (TIF) [file pgen.1010927.s002.tif]

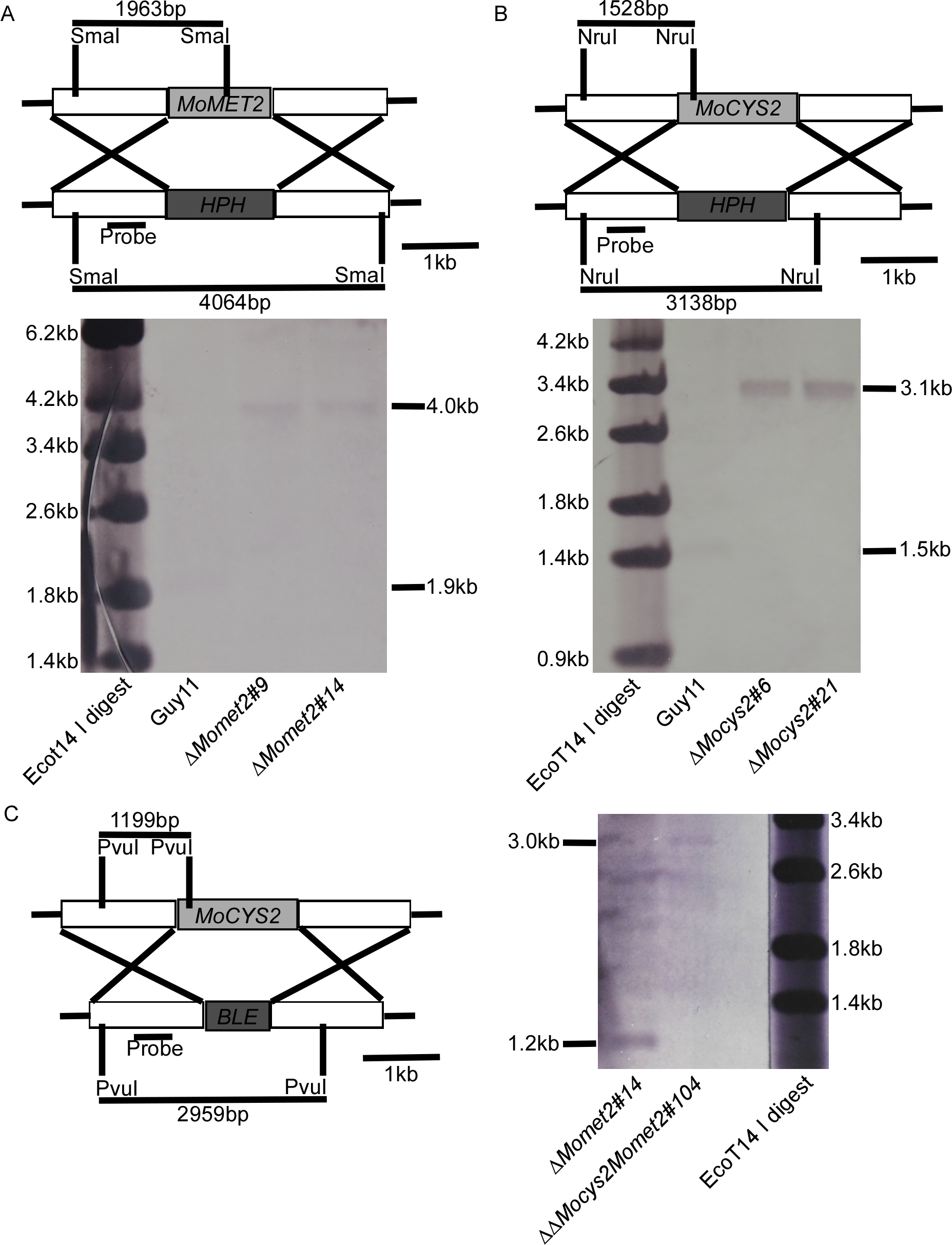

Supplement: S3 Fig — (A) Amino acid alignment of MGG_01469 and its ortholog met2 in Saccharomyces cerevisiae. (B) Amino acid alignment of MGG_14202 and its ortholog cys2 in Colletotrichum chlorophyti. (TIF) [file pgen.1010927.s003.tif]

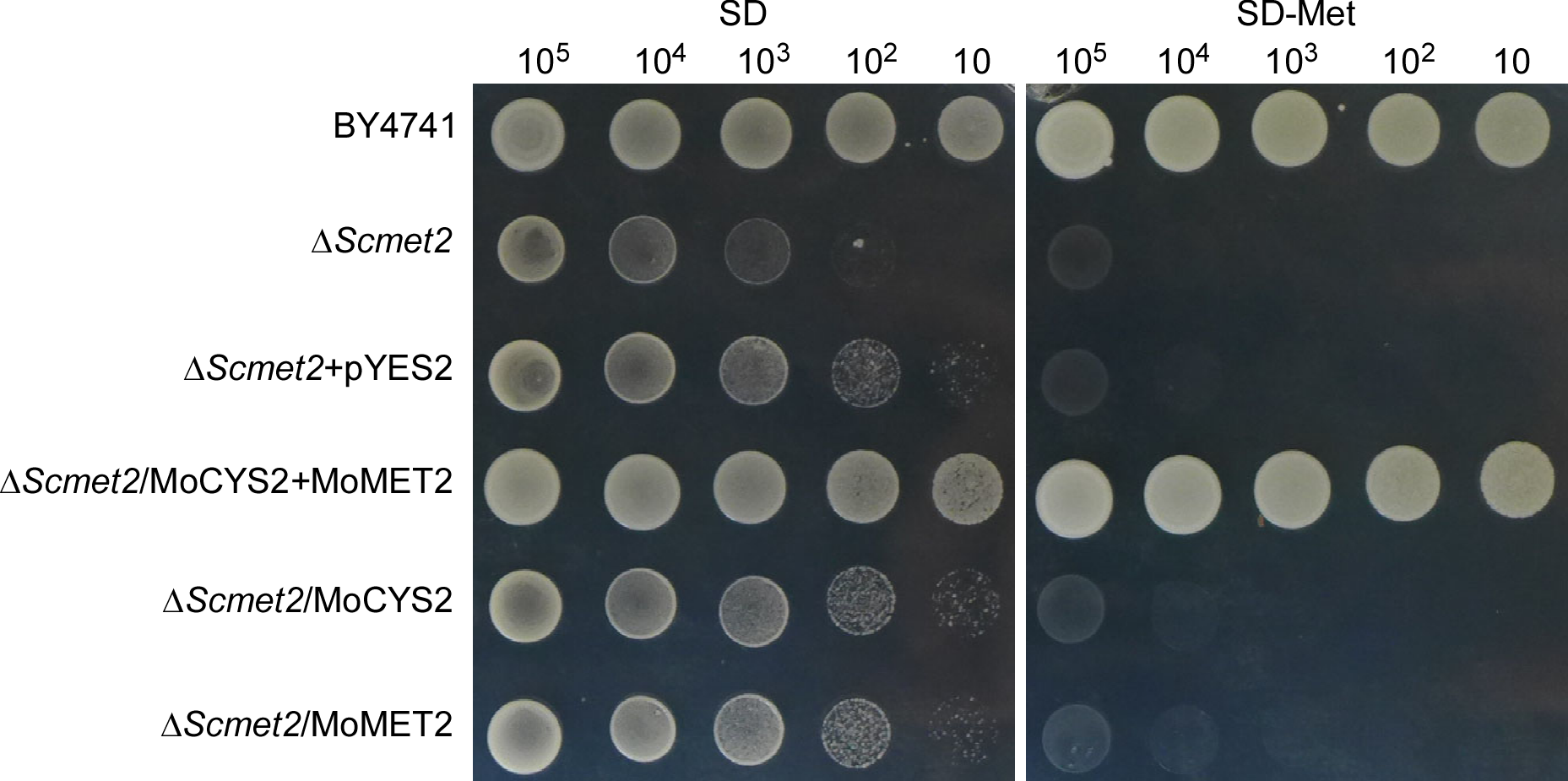

Supplement: S4 Fig — (A) Amino acid alignment of MGG_01469 and its ortholog met2 in Saccharomyces cerevisiae. (B) Amino acid alignment of MGG_14202 and its ortholog cys2 in Colletotrichum chlorophyti. (TIF) [file pgen.1010927.s004.tif]

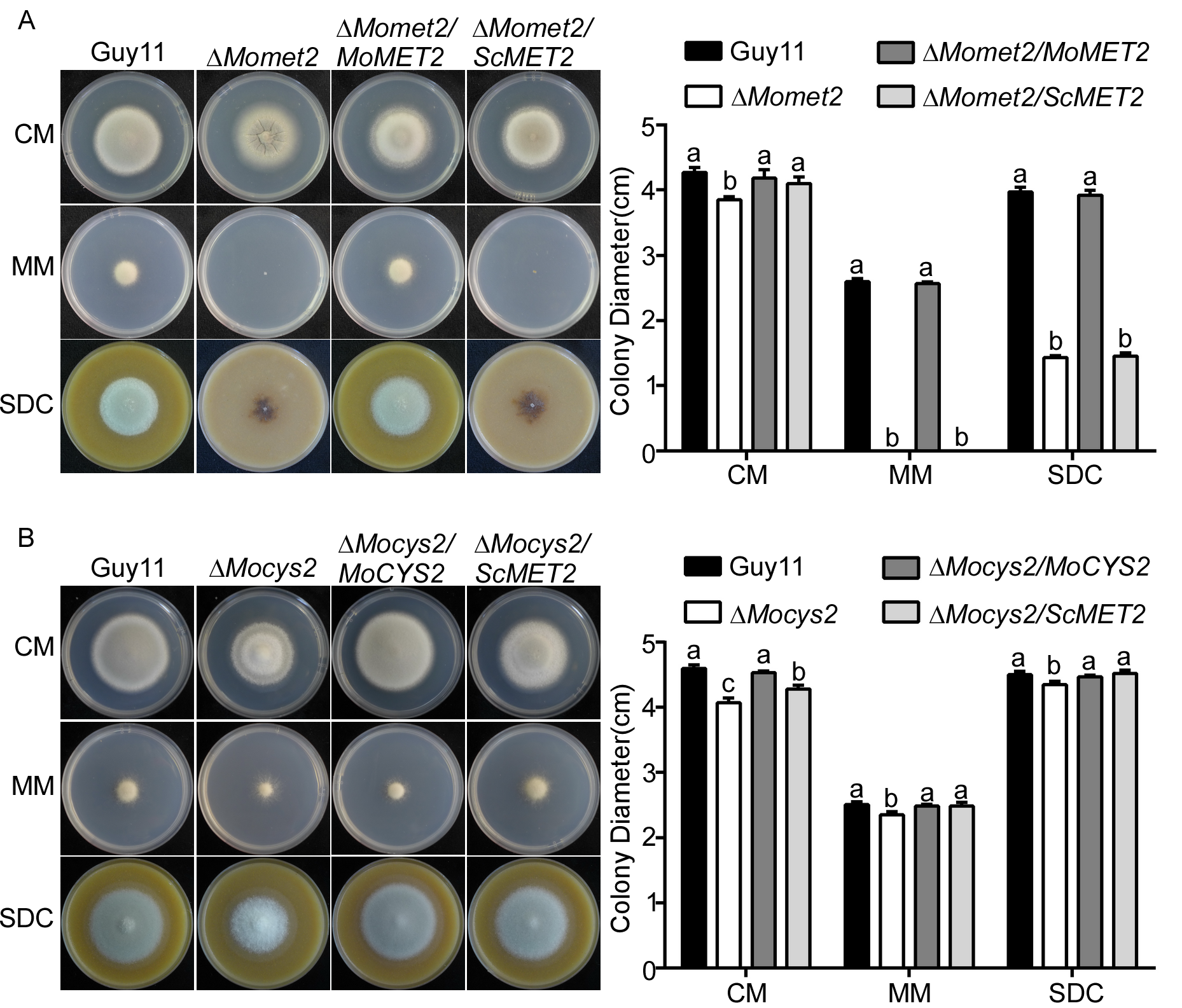

Supplement: S5 Fig — (A) Vegetative growth of Guy11, ΔMomet2, ΔMomet2/MoMET2, ΔMomet2/ScMET2 were grown on CM, MM, SDC. (B) Vegetative growth of Guy11, ΔMocys2, ΔMocys2/MoCYS2, ΔMocys2/ScMET2 were grown on CM, MM, SDC. Error bars represent SD and different letters indicate significant differences (P < 0.05) tested by one-way ANOVA with Duncan’s post hoc test. (TIF) [file pgen.1010927.s005.tif]

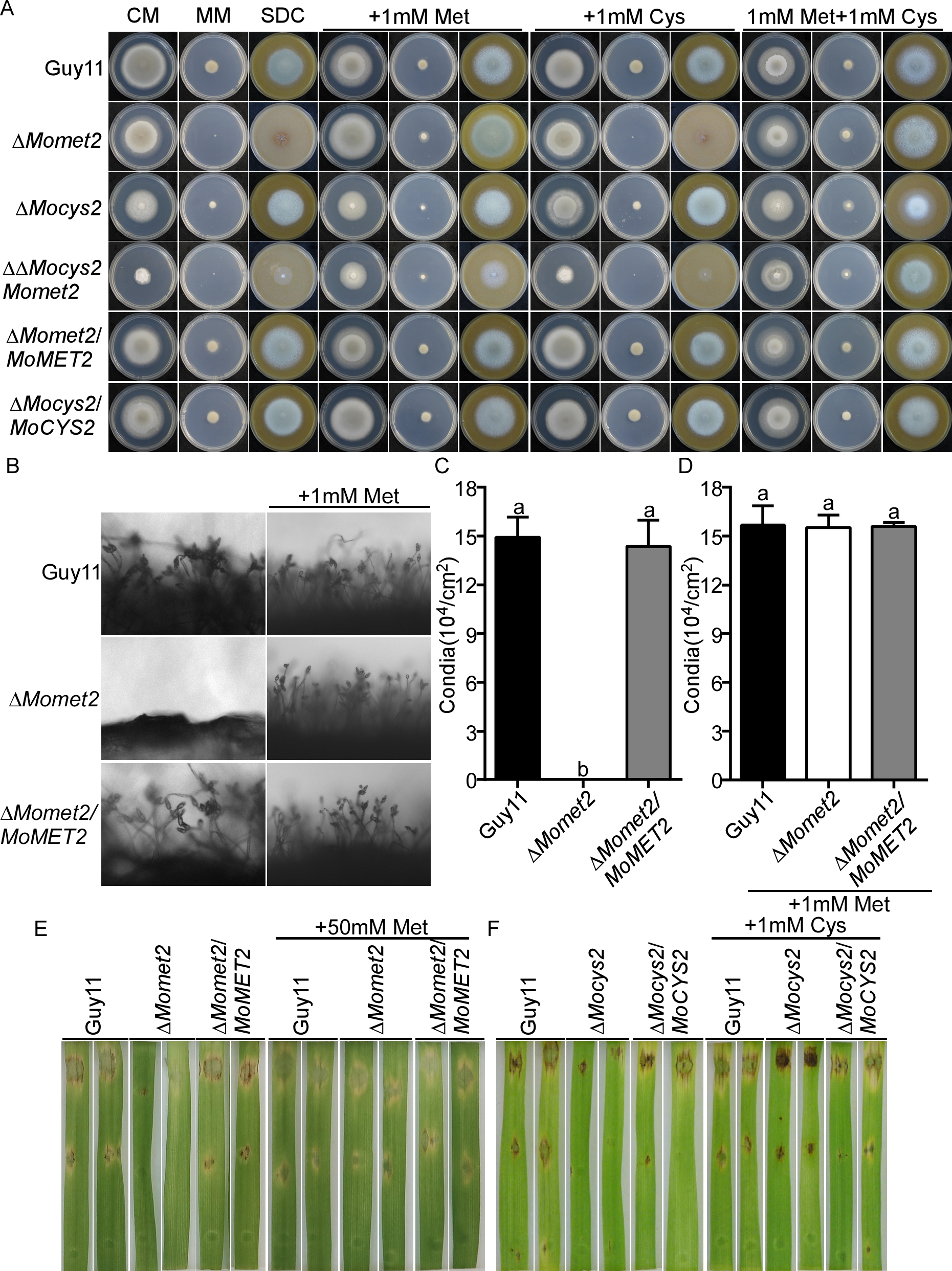

Supplement: S6 Fig — (A) Vegetative growth of the M. oryzae strains on CM MM and SDC supplemented with 1mM Met or 1mM Cys. (B) Conidia were observed under a light microscope after illumination for 24 h and photographed. (C, D) Statistical analysis of conidial numbers of the indicated strains. Error bars represent the SD and different letters indicate significant differences (P < 0.05) tested by one-way ANOVA with Duncan’s post hoc test. (E, F) Virulence analysis of the M. oryzae strains supplemented with 50 mM Met or 1 mM Cys. (TIF) [file pgen.1010927.s006.tif]

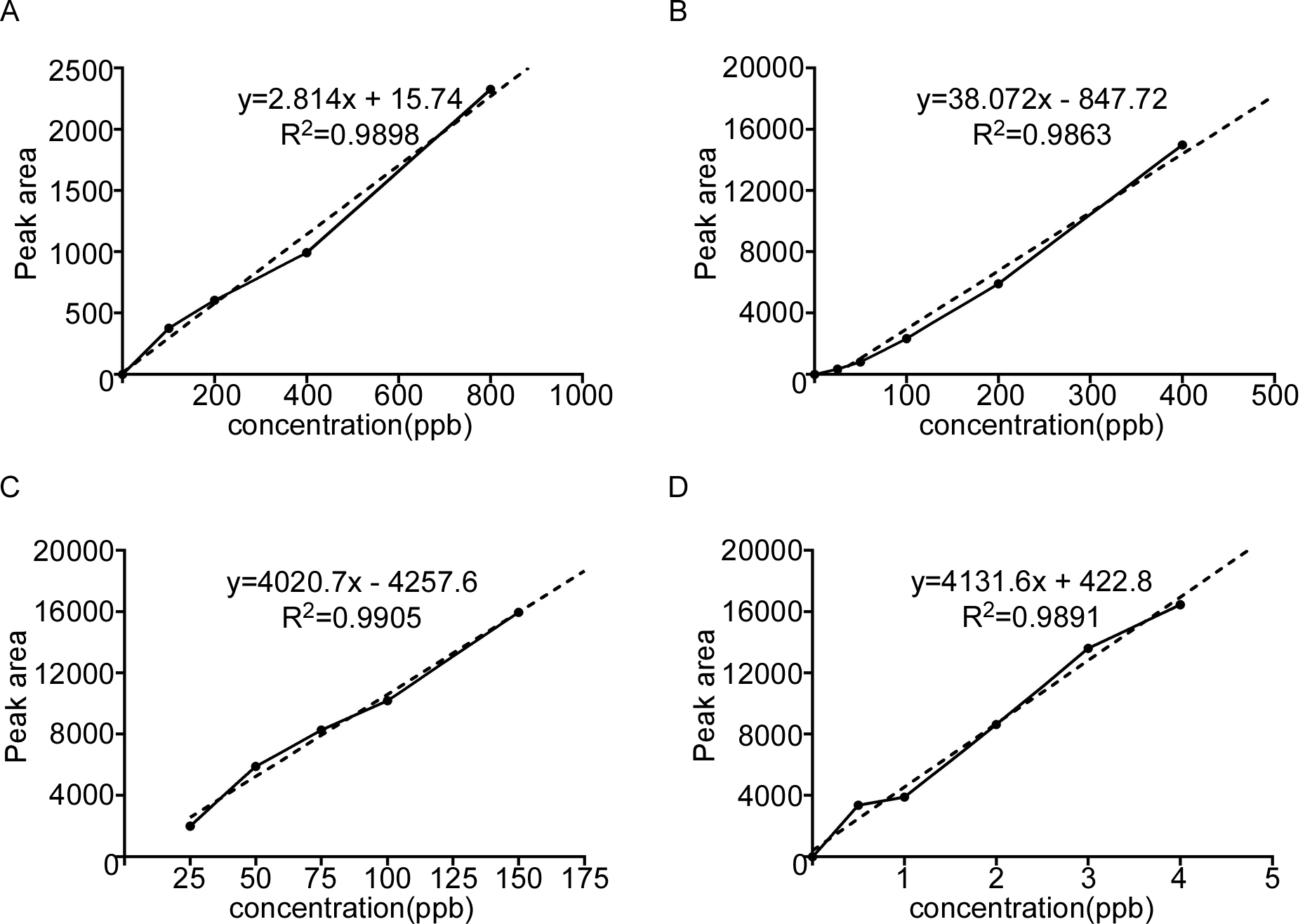

Supplement: S7 Fig — (A, B, C, D) Calibration curve of Met, SAM, dC and 5mC, respectively. (TIF) [file pgen.1010927.s007.tif]

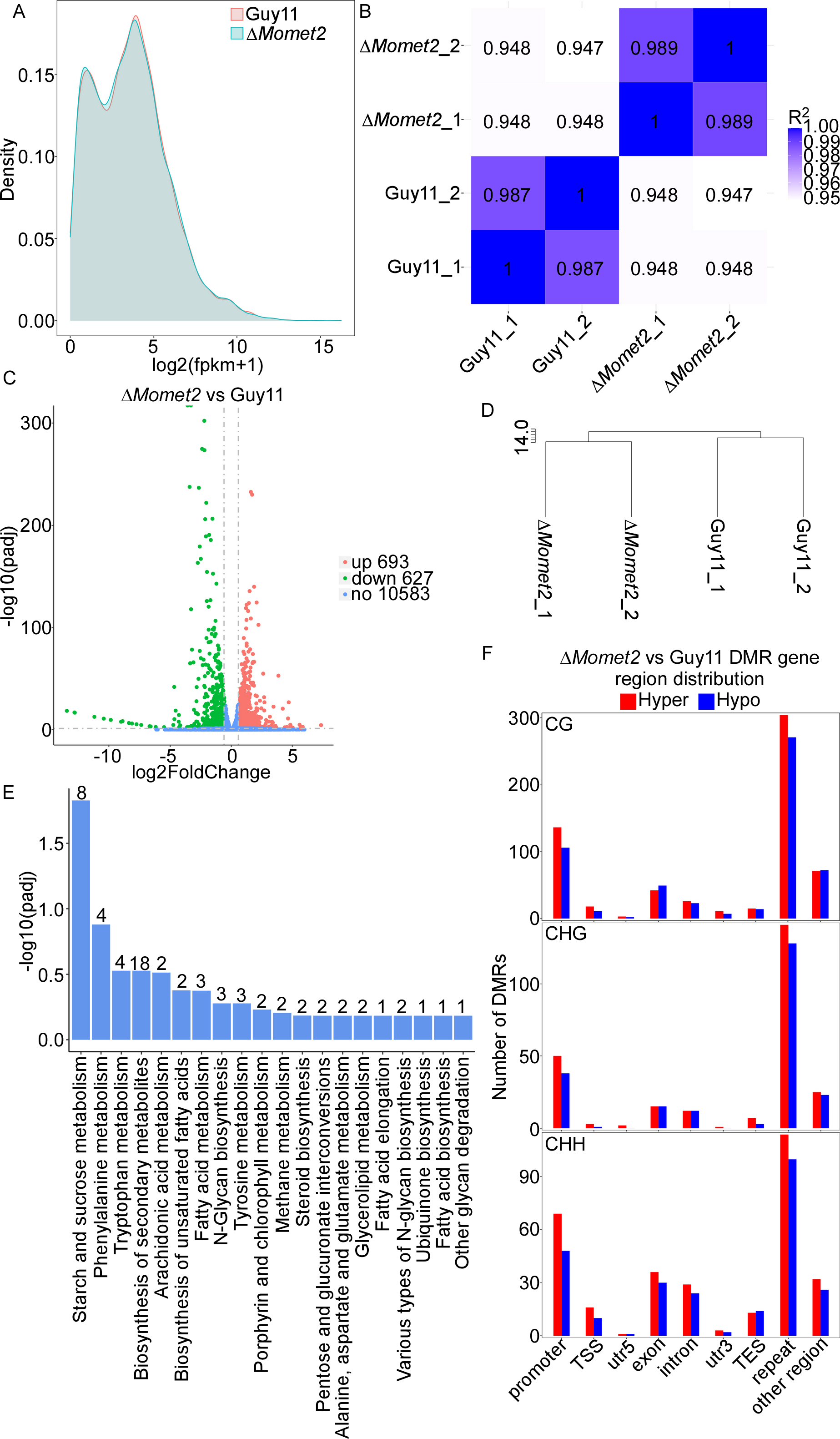

Supplement: S8 Fig — (A) Distribution of expression along the whole mRNA transcripts of M. oryzae detected in wild type strain and the MET2 deletion mutant. (B) The heatmap showing similarities exanimated in correlation analysis between Guy11 and ΔMomet2. (C) Volcano plot of. ΔMomet2 mutant gene expression pattern. (D) The dendrogram of sample clustering from Guy11 and ΔMomet2 WGBS. (E) KEGG analysis of ∆Momet2—down genes from RNA sequencing. (F) Hyper/Hypo number distribution in DMR anchoring region. (TIF) [file pgen.1010927.s008.tif]

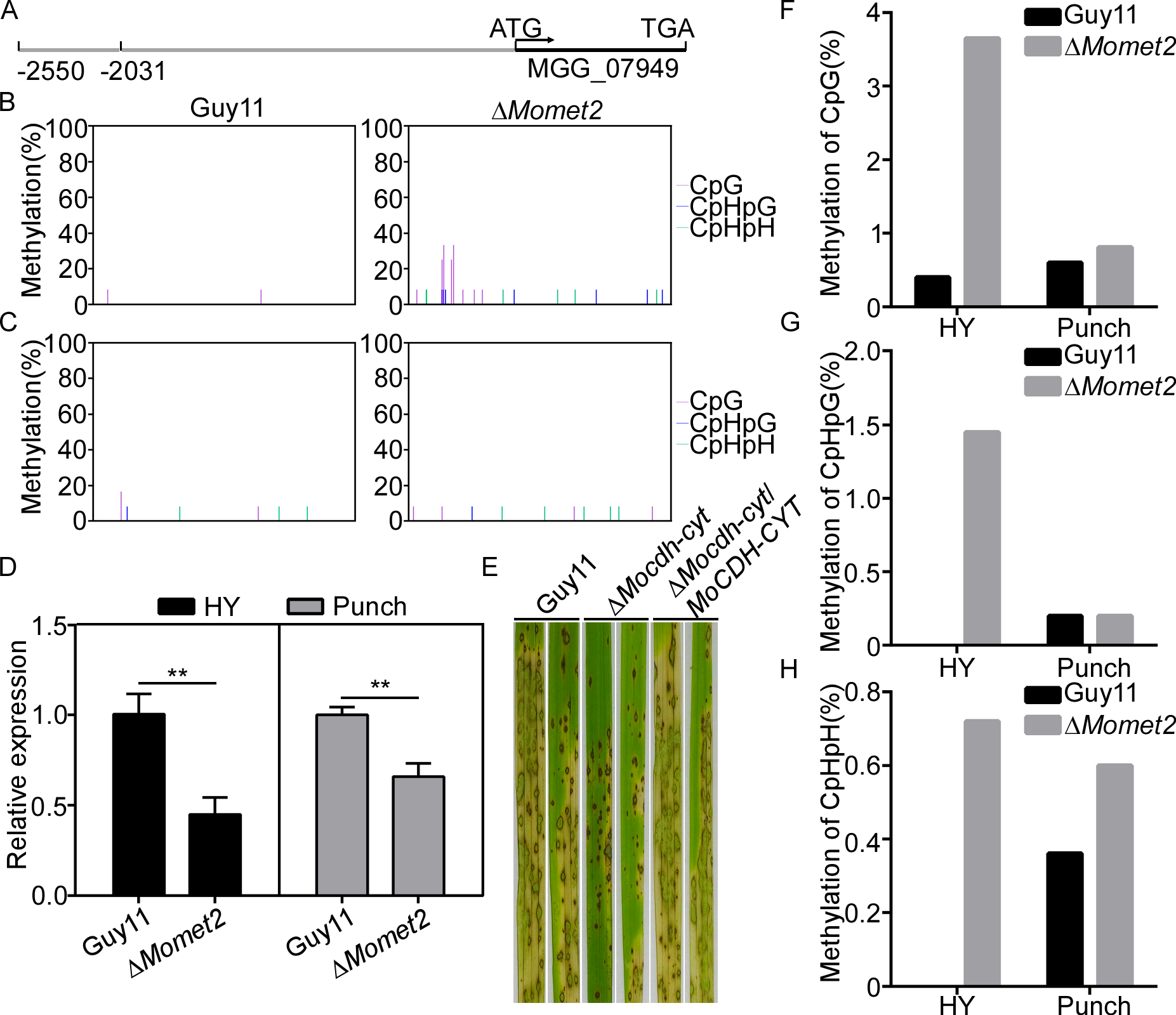

Supplement: S9 Fig — (A, B, C) Distribution of cytosine DNA methylation in three contexts in 519 bp of the MoCDH-CYT promoter region in the Guy11 and ΔMomet2 HY (B) and punched rice (C) as measured by bisulphite sequencing. Sequencing data were analyzed using Kismeth software. CpG, lilac; CpHpG, blue; CpHpHp, green. At least 12 clones were sequenced per sample. (D) The relative expression of MoCDH-CYT in different treatments. Error bars represent the SD (Student’s t test, *P < 0.05, **P < 0.01). (E) Pathogenicity test on rice leaves. (F, G, H) Methylation levels of CpG, CpHpG, and CpHpHp in the Guy11 and ΔMomet2. (TIF) [file pgen.1010927.s009.tif]

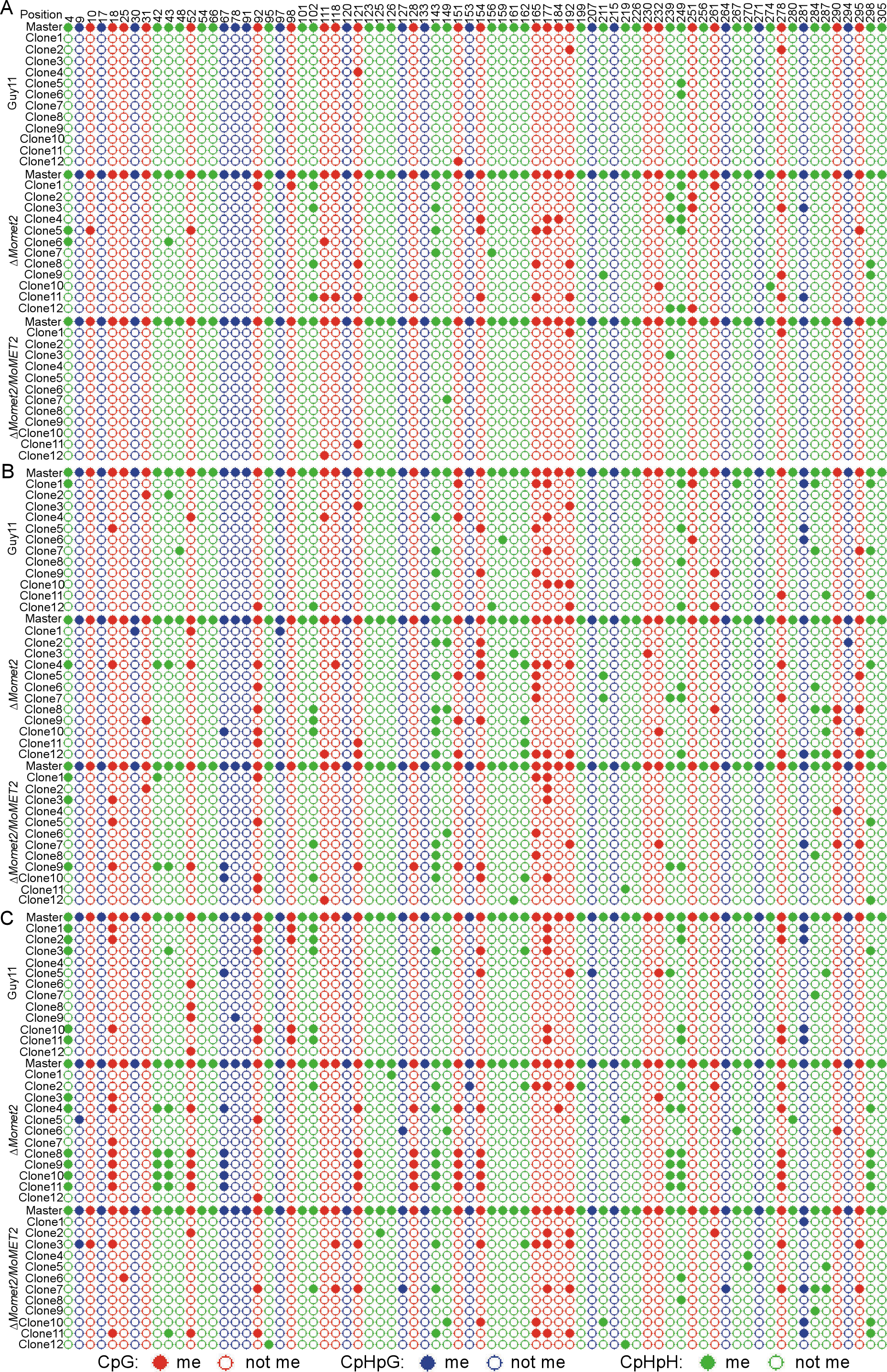

Supplement: S10 Fig — Bisulfite sequencing data of individual clones were submitted to the CyMATE program to analyze the methylated cytosines. Distribution of cytosine DNA methylation in three contexts in the MoGLIK promoter region in the Guy11, ΔMomet2 and the complemented mutant ΔMoMET2/MoMET2 HY (A) HY added 1 mM DTT (B) and punched rice (C) as measured by bisulfite sequencing. (TIF) [file pgen.1010927.s010.tif]

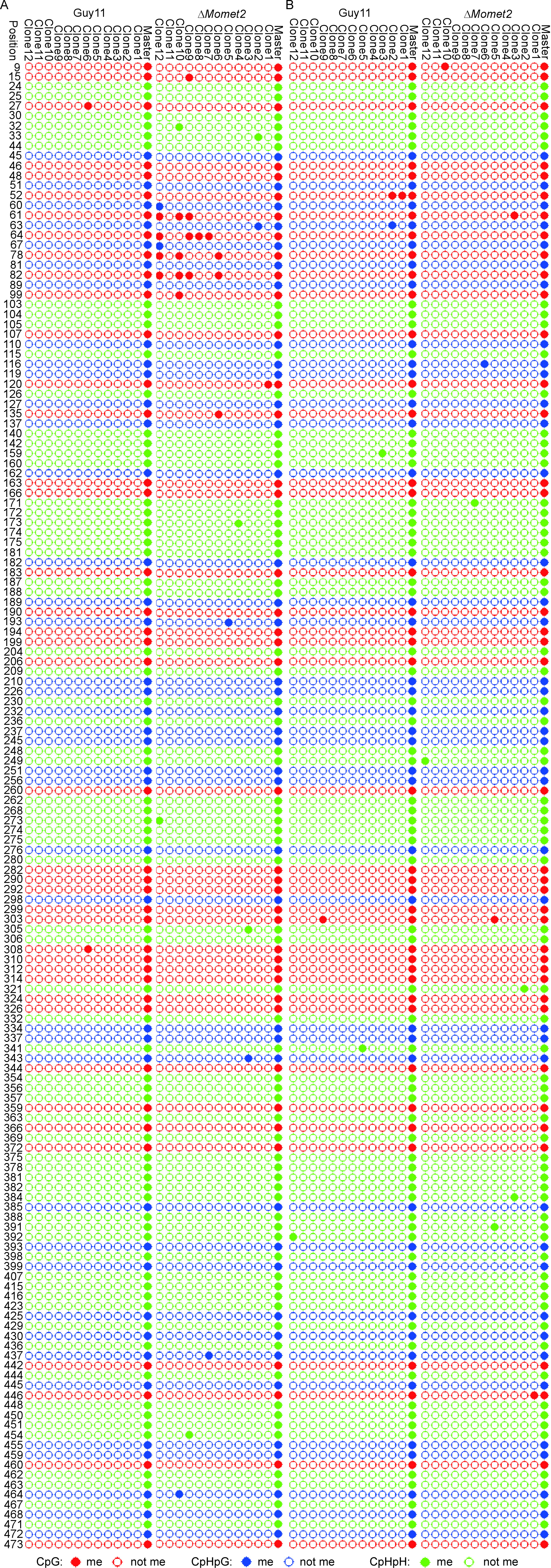

Supplement: S11 Fig — Bisulfite sequencing data of individual clones were submitted to the CyMATE program to analyze the methylated cytosines. Distribution of cytosine DNA methylation in three contexts in the MoCDH-CYT promoter region in the Guy11 and ΔMomet2 HY (A) and punched rice (B) as measured by bisulfite sequencing. (TIF) [file pgen.1010927.s011.tif]

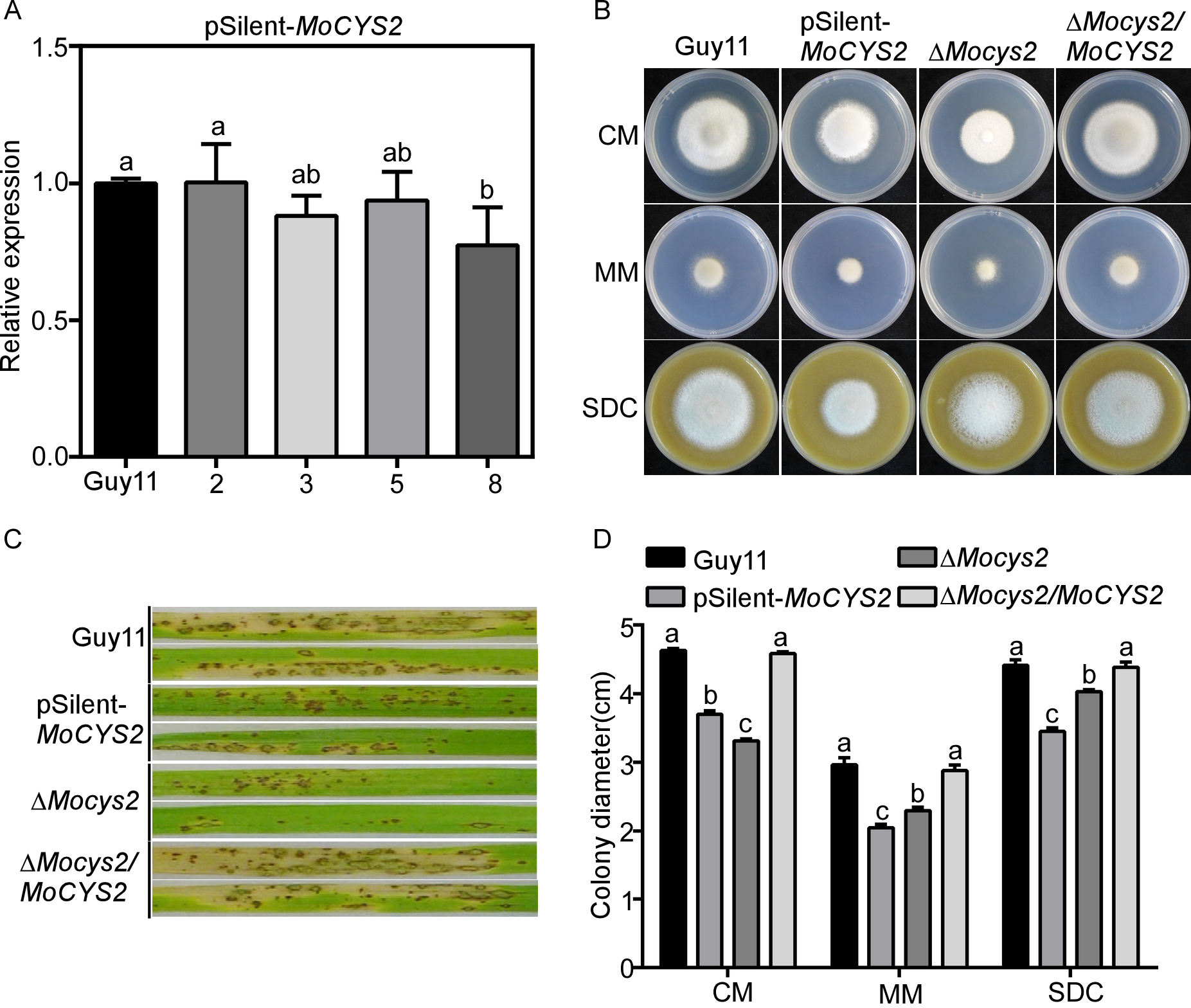

Supplement: S12 Fig — (A) The relative expression of MoCYS2 in different silenced strains. 2, 3, 5, 8, silenced strains. Error bars represent the SD and different letters indicate significant differences (P < 0.05) tested by one-way ANOVA with Duncan’s post hoc test. (B, D) Vegetative growth of the 8 silent stains on CM, MM, SDC. Error bars represent the SD and different letters indicate significant differences (P < 0.05) tested by one-way ANOVA with Duncan’s post hoc test. (C) Virulence analysis of the 8 silent stains on rice. (TIF) [file pgen.1010927.s012.tif]

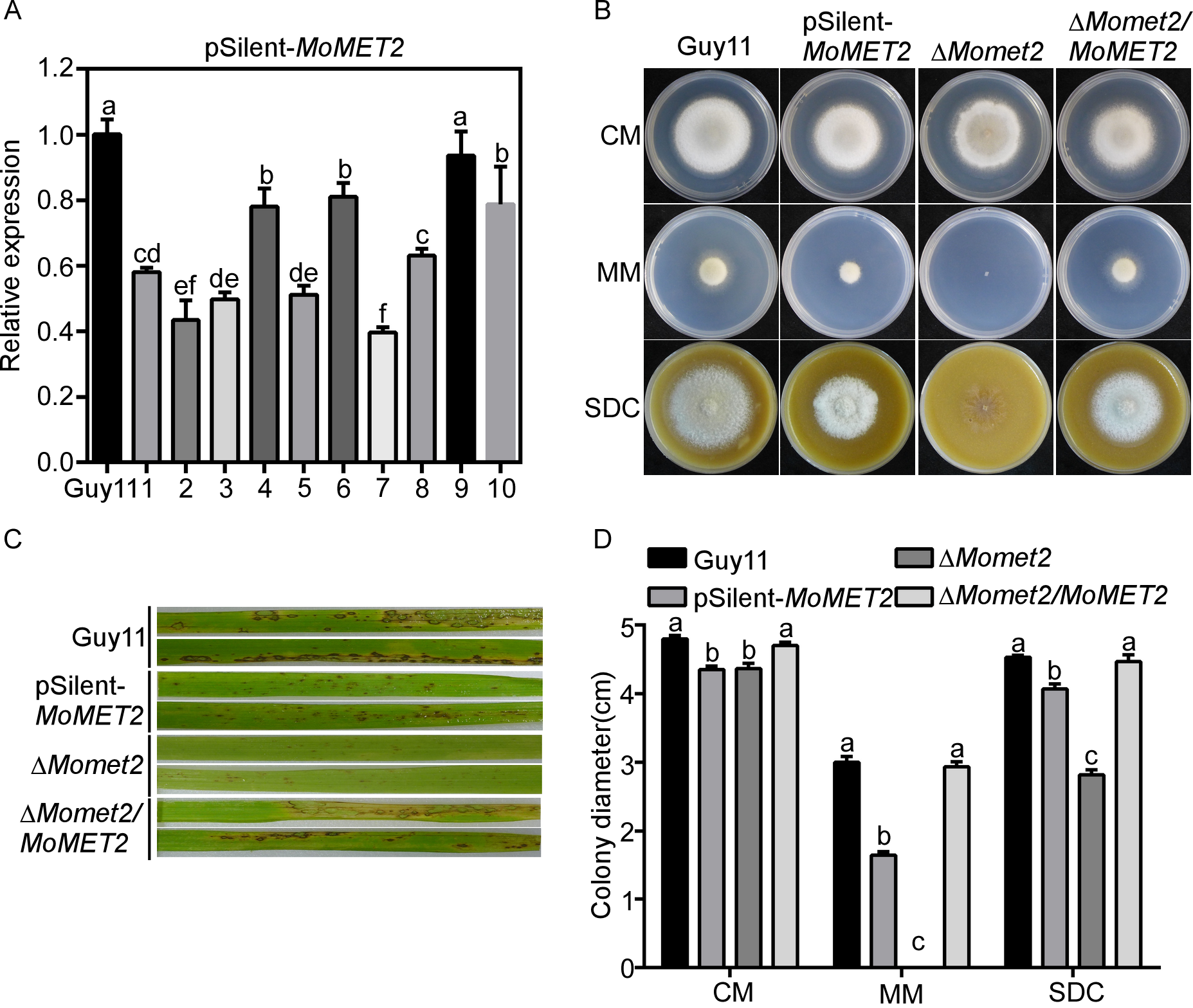

Supplement: S13 Fig — (A) The relative expression of MoMET2 in different silenced strains. 1–10, silenced strains. Error bars represent the SD and different letters indicate significant differences (P < 0.05) tested by one-way ANOVA with Duncan’s post hoc test. (B, D) Vegetative growth of the 7 silent stains on CM, MM, SDC. Error bars represent SD and different letters indicate significant differences (P < 0.05) tested by one-way ANOVA with Duncan’s post hoc test. (C) Virulence analysis of the 7 silent stains on rice. (TIF) [file pgen.1010927.s013.tif]

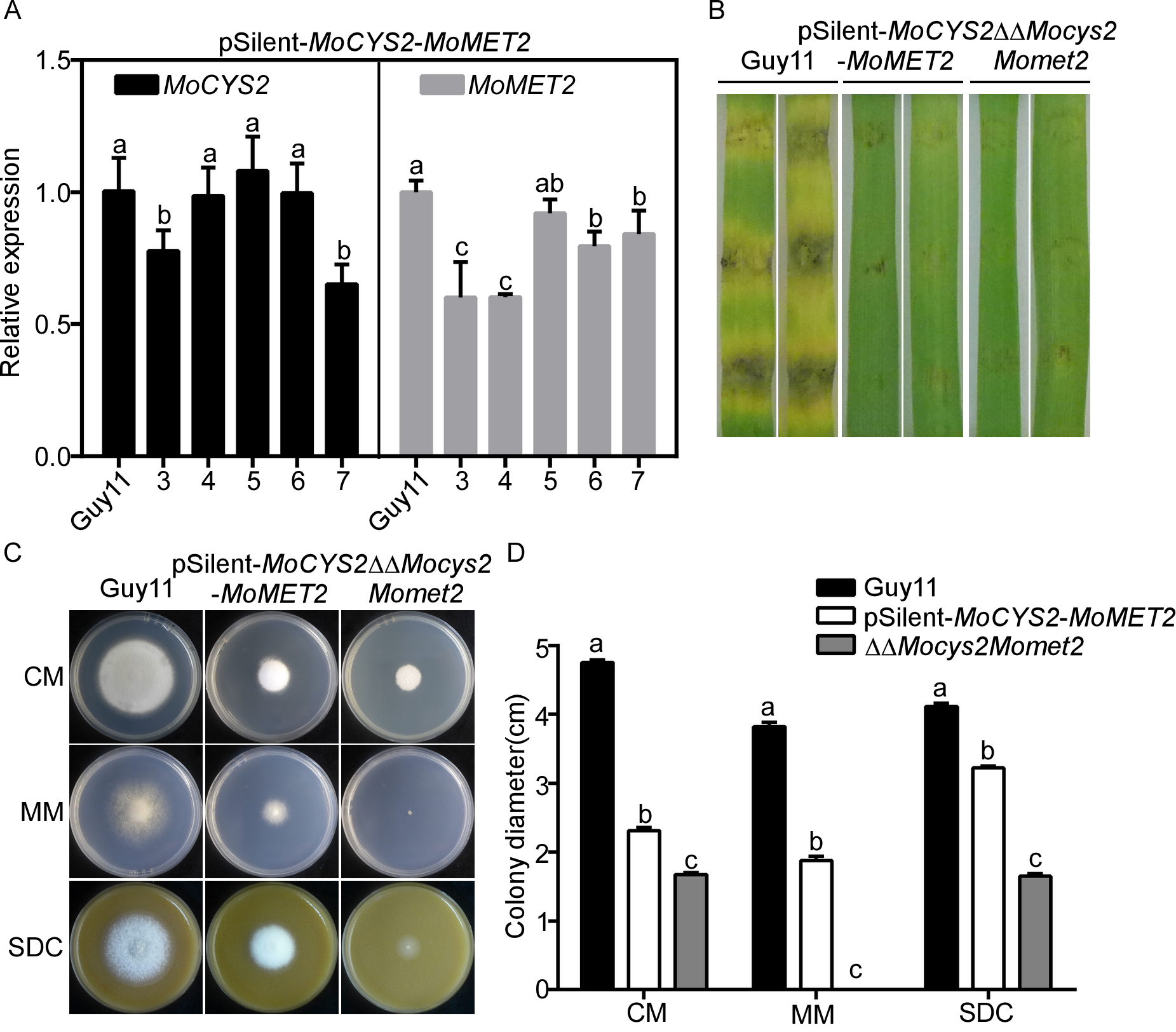

Supplement: S14 Fig — (A) The relative expression of MoCYS2 and MoMET2 in different silenced strains. 3–7, silenced strains. Error bars represent the SD and different letters indicate significant differences (P < 0.05) tested by one-way ANOVA with Duncan’s post hoc test. (B) Virulence analysis of the 7 silent mutants on barley. (C, D) Vegetative growth of the 7 silent stains on CM, MM, SDC. Error bars represent SD and different letters indicate significant differences (P < 0.05) tested by one-way ANOVA with Duncan’s post hoc test. (TIF) [file pgen.1010927.s014.tif]

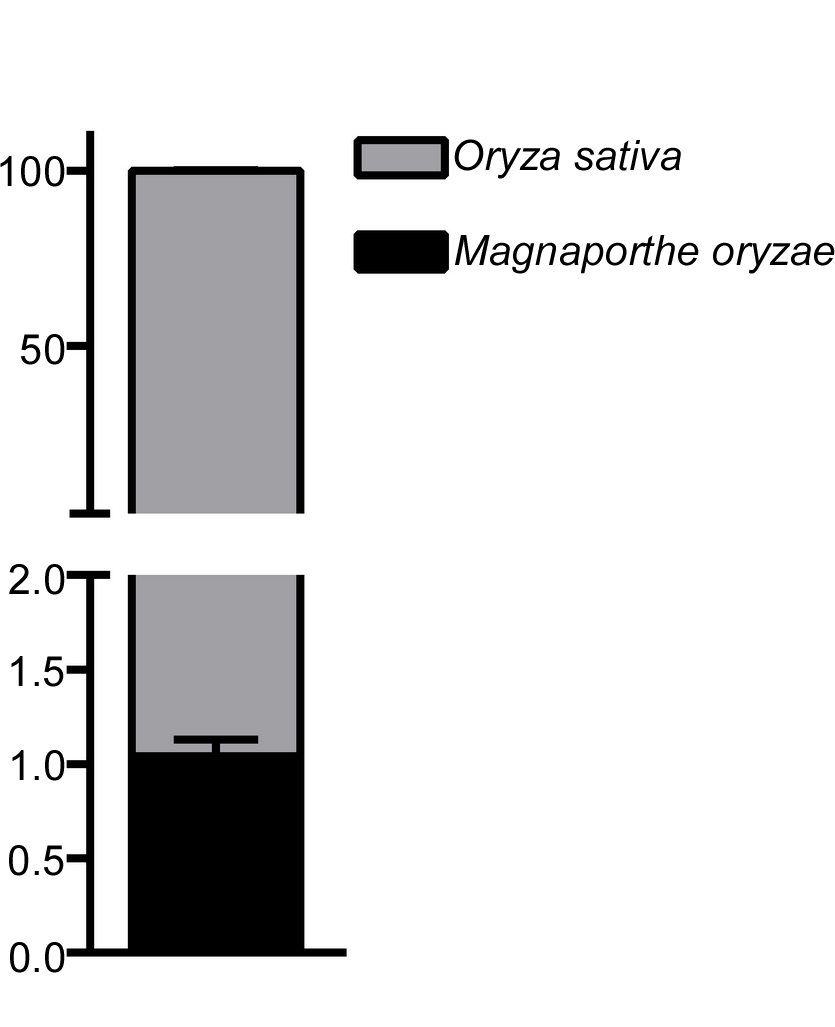

Supplement: S15 Fig — (TIF) [file pgen.1010927.s015.tif]
